# Somatostatin and Its Analogues as Second-Line Treatments in Non-Neoplastic Conditions

**DOI:** 10.3390/ijms27093816

**Published:** 2026-04-25

**Authors:** Argyrios Periferakis, Lamprini Troumpata, Ioannis Xefteris, Alexandros Kanellos Mavrokefalos, Aristodemos-Theodoros Periferakis, Konstantinos Periferakis, Ana Caruntu, Andreea-Elena Scheau, Christiana Diana Maria Dragosloveanu, Constantin Caruntu, Cristian Scheau

**Affiliations:** 1 Department of Physiology, The “Carol Davila” University of Medicine and Pharmacy, 050474 Bucharest, Romania; 2 Akadimia of Ancient Greek and Traditional Chinese Medicine, 16675 Athens, Greece; 3 Elkyda, Research & Education Centre of Charismatheia, 17675 Athens, Greece; 4 Pan-Hellenic Organization of Educational Programs (P.O.E.P.), 17236 Athens, Greece; 5 Department of Oral and Maxillofacial Surgery, “Carol Davila” Central Military Emergency Hospital, 010825 Bucharest, Romania; 6 Department of Oral and Maxillofacial Surgery, Faculty of Dental Medicine, Titu Maiorescu University, 031593 Bucharest, Romania; 7 Department of Radiology and Medical Imaging, “Foisor” Clinical Hospital of Orthopaedics, Traumatology and Osteoarticular TB, 021382 Bucharest, Romania; 8 Department of Ophthalmology, Faculty of Dentistry, The “Carol Davila” University of Medicine and Pharmacy, 020021 Bucharest, Romania; 9 Department of Ophthalmology, Clinical Hospital for Ophthalmological Emergencies, 010464 Bucharest, Romania; 10 Department of Dermatology, “Prof. N.C. Paulescu” National Institute of Diabetes, Nutrition and Metabolic Diseases, 011233 Bucharest, Romania

**Keywords:** somatostatin, octreotide, lanreotide, pasireotide, congenital hyperinsulinism, ocular pathologies, gastrointestinal bleeding, dumping syndrome, acute pancreatitis, portal hypertension

## Abstract

Somatostatin is a potent endocrine regulator and neurotransmitter, exerting predominantly inhibitory effects in different tissues of the body, via G-protein coupled receptors. Five such specific receptors have been identified, with different effects and tissue distribution. The multifaceted actions and effects of somatostatin make it useful as a potential therapeutical means in various pathologies; however, in clinical practice, somatostatin analogues, namely octreotide, lanreotide and pasireotide, are commonly used instead, due to their increased half-life and increased receptor selectivity, with pasireotide showing a more extensive receptor binding profile and high affinity for somatotastin receptor (SSTR) 5, which may prove effective in cases of resistance to first-generation analogues. Apart from their many uses in neoplastic pathologies, somatostatin analogues represent viable treatment choices in some ocular pathologies, congenital hyperinsulinism, gastrointestinal bleedings and portal hypertension, acute pancreatitis, and dumping syndrome. They have also been used in some cases, with varying degrees of success, in patients with post-surgical gastrointestinal and lymphatic fistulas, refractory chronic diarrhoea and polycystic kidney disease; many applications in paediatric patients have also been documented. The aim of this review is to present the applications of somatostatin and its analogues as alternative or second-line therapies, along with insights into their effectiveness and future potential.

## 1. Introduction

Somatostatin (SS) is a cyclic peptide originally identified as a very potent growth hormone (GH) inhibitor, with a number of different, albeit inhibitory, physiological actions [1,2]. It occurs in two forms called somatotropin release-inhibiting factors (SRIFs), comprising 14 and 28 amino acids, respectively, which are the result of post-translational modification of preproSRIF, a precursor molecule expressed in a number of tissues [3,4,5].

Somatostatin is able to inhibit adrenocorticotropic hormone (ACTH) release and thyrotropin (TSH) release in tumours secreting it [6]. This is only a fraction of its physiological effects, and its demonstrated roles expand to neurotransmitter, neuromodulator, endocrine hormone, and paracrine factor; a potential trophic factor role is under investigation [7,8].

This impressive array of functions and effects of somatostatin is made possible via the interaction of somatostatin with its receptors, called somatostatin receptors, which are G-protein coupled receptors [9]. Apart from morphofunctional similarities between the receptors, they are coded for by different genes, and their qualitative and quantitative tissue distribution is different [10,11,12,13,14,15,16,17,18,19,20,21,22,23,24,25,26].

The activation of SSTRs, either by SS itself or its analogues, leads to a variety of effects via different signalling pathways [27]; many of these effects are of therapeutic importance, both in neoplasias (e.g., [28,29,30,31,32,33,34,35,36,37,38,39,40]) and also in non-neoplastic pathologies, as will be explored in the following sections.

The widespread use of somatostatin analogues, in certain fields and treatment schemes, instead of the native hormone, is justified by the rapid degradation of somatostatin in the human body [27] by ubiquitous peptidases [41]. Moreover, somatostatin administration causes an unacceptable suppression of insulin secretion [42,43,44,45]. While it was discovered fairly rapidly that SS analogues were more effective in suppressing GH than somatostatin itself, it was difficult to understand the aetiology behind this phenomenon. Only when the existence of different receptors became apparent was it possible to identify the molecular basis behind the differential response to SS and its analogues [46].

The common pharmacodynamic property of all SS analogues that have been synthesized to date is that they mimic the actions of endogenous somatostatin. However, not all SS analogues exhibit the same affinity for all SSTRs. The principal somatostatin analogues in clinical practice today are octreotide, lanreotide, and pasireotide.

Octreotide was the first to be clinically developed and widely used in humans [47] and is a more potent suppressor of GH, insulin-like growth factor 1 (IGF-1), and insulin, compared to somatostatin. It has been used continuously for over 40 years [48]. It is a cyclic octapeptide with the same inhibitory properties as somatostatin but far more resistant to degradation and greater affinity for SSTR_1_ and SSTR_5_. Lanreotide is another octapeptide but with greater affinity for SSTR_1_. Pasireotide, the only widely-used 2nd generation SS analogue, has a completely different structure, and its greatest affinity is for SSTR_4_ [46,48,49,50,51,52].

Vapreotide is a cyclic octapeptide with a rapid elimination rate and short half-life, of approximately 10 min, with probable first-order kinetics. After its rapid metabolization, its metabolite is des-(Trp8-NH2)re vapreotide, which exhibits no affinity for SSTRs and is mostly biliary eliminated. It generally suppresses secretions both at the level of the stomach and of the pancreas [53]. Rat experiments also indicate a vasoconstrictive effect in portal hypertension [54]. Fairly recent animal experiments indicate that vapreotide is more potent in inhibiting growth hormone, gastric acid, and bicarbonate release, compared to other somatostatin analogues. While its safety profile has been well established in a series of clinical trials [53], its clinical uses have been far fewer compared to the other analogues.

Today, SS analogues are used as first-line treatments in certain types of neoplasias, including hepatocellular carcinoma and acromegaly, where their successful clinical use has been documented extensively [48,55,56,57]. Apart from these widespread uses, due to its ubiquitous inhibitory action [58], their application was attempted in a number of other pathologies, as an alternative or second-line treatment scheme.

In the majority of non-neoplastic conditions, SS analogues are not considered first-line therapies; they are used in cases where standard treatment does not elicit a sufficient response or first-line agents are not tolerated or are contraindicated. Also, there are cases where the pathophysiological mechanism justifies their use, such as the expression of specific receptor subtypes. As such, SS analogues are defined as second-line therapeutic options not only through the perspective of safety and cost-effectiveness, but also efficient clinical agents [59,60].

In the sections below, we present and critically discuss the available evidence on the most common applications of somatostatin and its analogues in non-neoplastic disorders.

## 2. SS Analogues in the Treatment of Congenital Hyperinsulinism

In neonates and infants, the most common cause for persistent hypoglycaemia is hyperinsulinemic hypoglycaemia [61]; this is attributable to a wide spectrum of causes, but is invariably associated with severe and permanent sequelae if left untreated [62]. Hyperinsulinic hyperglycaemia can be transient or permanent, and it is usually caused by congenital hyperinsulinism; it is a rare disease with an incidence of less than 1:50,000 live births and appears in two forms, focal and diffuse. Invariably, the cause of congenital hyperinsulinism is genetic, and prompt diagnosis and treatment are essential [63].

If focal lesions are identified, surgery is considered the main treatment option, especially in cases where the lesion can be completely resected and the expertise of the surgical team is appropriate [64,65]. In diffuse hyperinsulinism, medical treatment is the preferred management strategy, with near-total pancreatectomy being reserved for cases where all pharmacological management has failed [64,66], due to the risk of complications, especially concerning impaired glucose metabolism [67]. First-line pharmacological treatment is diazoxide, with intravenous dextrose and glucagon as additional therapeutic agents [61,68].

Considering the risks for total or near-total pancreatectomy and the potential for an unsatisfactory response to diazoxide treatment, alternative approaches have also been sought. The rationale for using SS analogues in congenital hyperinsulinism is their potential of inhibiting insulin secretion via activation of SSTR_2_ and SSTR_5_ on pancreatic β-cells, leading to a reduction in associated hypoglycaemia [69,70]. Adverse effects should also be taken into account; for example, to treat hypoglycaemia, intravenous glucose (dextrose) or alternatively, increased doses of glucagon may be administered as an acute therapy—this last is associated with a rare but severe adverse effect, called necrolytic migratory erythema skin rash (NME) [71,72]. Diazoxide itself is also associated with potentially severe adverse effects [73]. SS analogues represent second-line treatment choices (Table 1), both to prevent pancreatectomy or in patients where an operation has already been performed, or in cases when the administration of first-line pharmacological treatments is ineffective or cannot be supported.

Hyperinsulinemic hypoglycaemia in paediatric patients was first treated with SS and its analogues in the 1970s [74,75,76,77,78,79,80]. The usefulness of octreotide in managing congenital hyperinsulinism in paediatric patients had already been recognized since the late 1980s, even though at this point the limited existing data did not allow for a complete evaluation of its effectiveness [78,81,82,83,84,85,86,87,88]. In 1993, Thornton et al. [89] noted that resistance to therapy developed after some time, and there was a possible impairment of linear growth, which was, however, not enough to warrant therapy discontinuation.

In 2012, a study in France used monthly long-acting octreotide injections over a 6-month period. Based on questionnaires filled in by both the paediatric patients themselves and their parents, the quality of life of the patients improved significantly, and there were no major adverse effects [90]. In 2013, Yorifuji et al. [91] concluded that a continuous octreotide infusion would be effective in patients with focal congenital hyperinsulinism due to mutations in a K_ATP_ channel. It was noted that due to the potential for adverse effects, the octreotide dose should be kept to a minimum, and the patients should be monitored for potential β-cell neoplasia even in cases of spontaneous remission [91].

In 2017, the SCORCH study was performed in paediatric patients unresponsive to diazoxide, who had either diffuse or focal lesions, and their parents did not consent to surgical treatment. An infusion of subcutaneous octreotide was administered subcutaneously. In three patients, there was a clinically significant rise in blood glucose levels, and one achieved complete remission after 606 days. In general, the treatment scheme was well-tolerated [92].

In six patients with poor tolerance or insufficient response to other treatments, very long-acting lanreotide was administered for a mean period of over 40 months. All patients had previously received octreotide, and with one exception of a patient having already undergone surgery, all the other treatments were performed prior to surgery. Half of the patients achieved better glycaemic control, and the hypoglycaemic episodes were reduced [93]. One year earlier, in two patients presenting with hypoglycaemia 30 min after, and diagnosed with congenital hyperinsulinism, long-acting lanreotide acetate was administered, following a previous octreotide administration. The treatment had comparable results to pump therapy but was better tolerated and preferred by the patients. It was not known yet whether they had the focal or the diffuse form, and they were too young to undergo surgery [94]. Corda et al. [95] reported the treatment of four infants, with poor response to previous drug and nutritional therapy. They concluded that during early infancy, long-acting lanreotide represents a viable alternative to surgery and can facilitate patient discharge from the hospital; no acute gastrointestinal adverse effects were noted. However, further data on the safety profile of lanreotide for such cases need to be gathered [95]. In a multicentre study, involving patients from different countries, close to 50% developed adverse effects, but only in very few were they enough to cause cessation of treatment [96]. It was noted that liver enzyme monitoring was necessary in such cases—especially for short-acting SS analogues—and SS analogue treatment should be avoided in cases with an increased risk of necrotizing enterocolitis. Moreover, monthly lanreotide injections were usually sufficient to maintain euglycemia [96]. The indication of SS analogues in treating neonates and infants should consider the safety of administration, especially with regard to potential complications, such as necrotizing enterocolitis, gastrointestinal intolerance, gallbladder sludge or cholelithiasis, as well as alterations of liver enzyme levels [97,98,99]. Prolonged treatment also raises concerns regarding the necessity of growth monitoring [100]. While these agents may help delay or even avoid extensive pancreatic surgery, the risk–benefit assessment should account for these potential complications, and close monitoring should be mandated [70].

Among the earliest uses of pasireotide in the treatment of congenital hyperinsulinism was that of a 38-week-old boy, unresponsive to diazoxide, with diffuse β-cell hyperplasia. This very severe form of congenital hyperinsulinism was due to a homozygous ABCC8 mutation. High doses of octreotide were administered, along with a continuous glucagon infusion and high carbohydrate intake. Long-acting lanreotide replaced octreotide to reduce the frequency of injections, but even so, due to continuous hypoglycaemia, partial pancreatic resection was performed [101]. In an attempt to avoid a second surgery, pasireotide was used, in twice-daily subcutaneous injections, later increased to four injections per day. Blood glucose levels improved but still fluctuated considerably. Being at the lower limit of the desired target range, this required a second and then a third partial pancreatectomy. Pasireotide administration was stopped after the third surgery [101].

Pasireotide was also used to treat two male twins of a mother with congenital hyperinsulinism; they received diazoxide, which was ineffective. Octreotide was used up to the maximum dose without effect. From day 92, pasireotide was administered subcutaneously, continuously via a pump, starting with 1000 μg/m^2^/day, and increased up to 1070 μg/m^2^/day and 1200 μg/m^2^/day for the first and second infant, respectively [102]. Both children were diagnosed with the diffuse form of congenital hyperinsulinism. At the age of 12 months, the long-acting form of pasireotide was used instead of the pump, administered once a month subcutaneously, along with diazoxide treatment. Due to a good glycaemic control, subtotal pancreatectomy was avoided [102].

**Table 1:** Clinical trials and research involving SS analogues in the treatment of congenital hyperinsulinism, arranged in chronological order.

Trial Type	Patients	Controls	Length of Trial	Dosage	Result	Year	Reference
NR	24 paediatric patients with no response to diazoxide	0	Varied (some less than 1 w)	Different doses of octreotide at different intervals depending on patient response	25% of patients avoided surgery, and a good response for most of the other patients	1993	[89]
NR	2 paediatric patients	0	>3 y	30 mg long-acting lanreotide sc once per month	Comparable to pump therapy but better tolerated	2011	[94]
NR	6 paediatric patients with previous octreotide treatment	0	40.8 m (mean)	60 mg/m very long-acting lanreotide sc increased to 90–129 mg/m depending on treatment response	Half of the patients achieved better results compared to previous therapy	2012	[93]
NR	10 paediatric patients unresponsive to diazoxide	0	6 m	30 mg long-acting octreotide sc once per month	Quality of life of patients was improved, and no significant adverse effects were noted	2012	[90]
NR	15 patients unresponsive to diazoxide	0	varied	Different doses of octreotide for different durations depending on the patient	Continuous subcutaneous octreotide infusions can lead to remission and is at least comparable to other available treatments	2013	[91]
NR	28 diazoxide unresponsive patients	0	varied	5–30 μg/kg/d octreotide as a continuous sc infusion	Treatment with octreotide is effective and usually presents with tolerable adverse effects	2014	[100]
NR (SCORCH Study)	5 paediatric patients with either diffuse or local lesions	0	varied	5–25 μg/kg/day octreotide continuous sc infusion	Clinically significant rise in blood glucose levels in 3 patients	2017	[92]
NR	4 infants with insufficient response to nutritional and drug treatment	0	varied	Different dosages and regimen of long-acting lanreotide depending on the patient	Long-acting lanreotide is a viable alternative to surgery for early infants	2017	[95]
NR	27 patients with poor response or tolerance to diazoxide	0	18 m (mean)	Different doses of lanreotide at different intervals depending on the patient	Monthly lanreotide injections were sufficient to maintain euglycemia	2018	[96]
NR	1 infant (38 w) with a severe diffuse form	0	2 m	Various, starting with 2 daily sc injections of pasireotide 0.15 mg	Better results in glycaemia control than lanreotide and octreotide but still not enough to avoid near-total pancreatectomy	2021	[101]
NR	2 twin infants with a diffuse form	0	~12 m	Different doses, starting from a continuous infusion and progressing to monthly sc injections of pasireotide	Good glycaemic control and avoidance of subtotal pancreatectomy	2024	[102]

NR = non-randomised, w = week, m = months, y = years, sc = subcutaneously.

## 3. SS Analogues in Refractory Diabetic Retinal Disease

Alterations in the GH/IGF-1 axis have been implicated in diabetes-related metabolic and vascular complications, including diabetic retinal disease [103,104,105,106,107,108,109,110,111]. While native somatostatin can modulate these pathways, its use in diabetic patients is impractical due to its short half-life [112]; initial experiments using octreotide—which is long-acting and therefore requires fewer injections—did not have encouraging results [113,114]. While there is evidence supporting the idea that SS can contribute towards maintaining euglycemia [115,116], even the use of octreotide, with its longer half-life, would require 3–4 daily injections, and potentially a major redesign of the pharmacological scheme for diabetic patients [117]. Nevertheless, SS analogues have been clinically tested for their potential effects on some of the ocular comorbidities associated with diabetes mellitus, such as diabetic retinopathy and diabetic macular oedema (DME) (Table 2).

Diabetic retinal disease is a major cause of visual impairment, while proliferative diabetic retinopathy and DME represent the main causes of vision loss in patients with diabetes mellitus [118,119]. More broadly, the clinical expression may be impacted by individual anatomical characteristics, which should be considered when assessing disease burden and therapeutic response [120,121]. While there are a number of treatment schemes [122], both pharmacological and interventional, there are patients with resistant forms of this disease [123]. In this context, SS analogues were analysed as potential second-line treatment options.

A feasibility study was conducted in 1989 by Hyer et al. [124], who administered octreotide in both diabetic patients with retinopathy and healthy patients, noting that it suppressed growth hormone-release hormone (GHRH)-stimulated growth hormone (GH) release in both groups, albeit not completely. The reduction of serum IGF-1 levels was believed to potentially be of significance in arresting the progression of diabetic retinopathy.

A randomised study was conducted by Kirkegaard et al. [125], who observed no effect in patients with early diabetic retinopathy. Similar results were obtained by the uncontrolled study of Grant et al. [126], who determined that octreotide was inefficient in preventing severe non-proliferative and “non-high risk” proliferative diabetic retinopathy. On the contrary, Mallet et al. [127] in 1992 proved in their uncontrolled study with four patients that octreotide can be efficient in the revascularization of the retina in cases of proliferative diabetic retinopathy unresolved by panretinal photocoagulation; another smaller study involving just two patients was performed later by Efthymiou et al. [128].

Following up on these results, the treatment of euthyroid diabetic patients with octreotide appears to be able to delay the progression of advanced retinopathy and postpone laser photocoagulation [129]. A subsequent study by Boehm et al. [130], including both type 1 and type 2 diabetics, confirmed the efficacy in controlling vitreous haemorrhages in patients with high-risk proliferative diabetic retinopathy after laser photocoagulation. This study was important because it was the first long-term study of SS analogue use in this pathology. Recently, octreotide LAR in association with intensive insulin was tried in Japan, in a patient who was suffering from type 2 diabetes mellitus complicated with diabetic proliferative retinopathy, nephropathy, and multiple peripheral neuropathies, and coexisting active acromegaly due to a pituitary adenoma. This led to improvement of visual acuity with no apparent progression of diabetic retinopathy [123].

SS itself was compared to brimonidine and placebo treatment in a prospective randomised long-term trial and was proven to be effective in retinal vascular dilation induced by long-term topical neuroprotection, although further studies are needed to fully quantify the effect and elucidate the underlying mechanisms [131]. Finally, lanreotide has also been tried in diabetes-associated ocular problems. In 2008, in a case study involving two female patients with poorly-controlled diabetes mellitus type 2 and diabetic macular oedema, where previous photocoagulation had been performed, lanreotide autogel was administered thrice per month for 12 months, and proved an effective treatment alternative in patients with persistent diabetic macular degeneration, also improving their quality of life [132]. An earlier case study, on one patient, using octreotide had also proved its therapeutic potential in a similar situation [133]. The most recent study involved lanreotide administration in 18 patients with persistent diabetic macular oedema, of whom five dropped out of the study due to the adverse effects of the drug.

The beneficial use of lanreotide in diabetic macular oedema was recently confirmed by Fernandez-Lopez et al. [134], in diabetic patients where diabetic macular oedema was persistent and refractory to treatment, although the existence of adverse effects, due to which five patients left the study, was noted.

Overall, clinical data on the use of SS analogues in diabetic retinal disease are heterogeneous, and the results are mixed. Some studies suggest a benefit in patients with persistent diabetic retinopathy or DME, but the evidence is limited and does not support their use over current therapies such as anti-VEGF-based approaches, which are well-established [135]. To this effect, SS analogues may be regarded only as potential second-line or adjunctive treatments in selected refractory cases.

**Table 2:** Clinical trials and research involving SS and its analogues in the management of diabetic macular degeneration, arranged in chronological order.

Trial Type	Patients	Controls	Length of Trial	Dosage	Results	Year	References
NR	9 patients with insulin-dependent diabetes	6 age-matched healthy patients	14 w (median)	50 μg octreotide sc 3 times per day and gradual dose increase (different max dose depending on patient) & continuous infusions in some patients	Potential benefit in diabetic retinopathy due to IGF-1 levels reduction	1989	[124]
R	11 patients with early diabetic retinopathy and type 1 (insulin resistant) diabetes mellitus	9 patients with early diabetic retinopathy and type 1 (insulin resistant) diabetes mellitus	1 y	50–400 μg octreotide sc per day (increase of 50 μg every second day up to 200 or 400, as needed)	No effect in early diabetic retinopathy	1990	[125]
NR	4 patients with proliferative diabetic retinopathy unresolved by panretinal photocoagulation	0	6–20 m	400 μg octreotide sc per day	Revascularization in the retina stopped/regressed	1992	[127]
NR	8 patients with severe non-proliferative and “non-high risk” proliferative diabetic retinopathy	8 patients with severe non-proliferative and “non-high risk” proliferative diabetic retinopathy	15 m	600–3000 μg octreotide sc or continuous infusion	Inefficiency in preventing severe non-proliferative and “non-high risk” proliferative diabetic retinopathy	1996	[126]
R	11 patients with severe non-proliferative and “non-high risk” proliferative diabetic retinopathy	12 patients with severe non-proliferative and “non-high risk” proliferative diabetic retinopathy	15 m	200–5000 μg octreotide sc per day/conventional diabetic treatment	Delay of progression of diabetic retinopathy in euthyroid patients; 27% ocular disease incidence in test group versus 42% in the control group	2000	[129]
R	9 patients with high-risk proliferative diabetic retinopathy after full scatter laser coagulation	9 patients with high-risk proliferative diabetic retinopathy after full scatter laser coagulation	3 y (max.)	100 μg octreotide (verum) sc tid	Reduction of risk for dense vitreous haemorrhages and vitreoretinal surgery	2001	[130]
NR	1 patient with type 1 diabetes mellitus with cystoid diabetic macular oedema	0	1 y	20 mg octreotide LAR im once every 4 w	Increased visual acuity and disappearance of cystoid changes	2004	[133]
NR	2 patients with poorly-controlled diabetes mellitus type 2 and persistent bilateral cystoid macular oedema	0	1 y	90 mg lanreotide autogel sc every 4 w	Reduction of cystoid changes and foveal thickness (and subsequently improved mental health)	2008	[132]
R	297 patients with type 2 diabetes mellitus with no or early in 1:1:1 randomization (104:85:108)		96 w	SS 0.1% 2 t/d topical administration (1st group); brimonidine 0.2% 2 t/d topical administration (2nd group); placebo 2 t/d topical administration (3rd group)	Topical treatment with either brimonidine or somatostatin causes retinal arteriolar and venular dilation in patients with type 2 diabetes and pre-existing early DR	2019	[131]
NR	1 patient with type 2 diabetes mellitus, proliferative diabetic retinopathy and diabetic macular oedema related to active acromegaly	0	1 m	20 mg octreotide-LAR im once for 1 month	Improvement of visual acuity with no apparent progression of diabetic retinopathy	2022	[123]
NR	18 patients with diabetes mellitus and persistent macular oedema resistant to conventional diabetic treatment	0	1 y	90 mg lanreotide sc once every 4 weeks	Lanreotide significantly reduces diabetic macular oedema in patients refractory to other treatments	2025	[134]

NR = non-randomised, R = randomised, w = week, m = months, y = years, im = intramuscular, sc = subcutaneously, t = times, IGF-1 = insulin-like growth factor 1, LAR = long-acting release.

## 4. SS and Its Analogues in Graves Orbitopathy (Thyroid Eye Disease) Management

Graves orbitopathy, also known as thyroid eye disease (TED), is an autoimmune disease, which is a frequent comorbidity in patients with hyperthyroidism [136,137,138,139]; however, about 10% of patients are euthyroid or even hypothyroid [140]. It causes swelling of the orbital tissue, leading to disfigurement, diplopia, and occasionally visual loss [141]. Like other ophthalmic disorders, ocular surface alterations may contribute to symptomatology and should not be disregarded in the overall assessment [142,143,144]. TED is characterized by lymphocytic infiltration and oedema of the retrobulbar tissues, resulting in marked swelling of extraocular muscles and orbital fat. The rising retrobulbar pressure interferes with venous drainage and causes lid swelling and exophthalmos [136,145], potentially leading to loss of visual function if the pressure rises sufficiently to damage the optic nerve [146]. In patients with TED, the first approach is to control thyroid function; cessation of smoking is recommended if applicable [147,148], and frequent follow-ups are required. Regarding pharmacological intervention, local or systemic glucocorticoids, local cyclosporine, teprotumumab, tocilizumab, rituximab, mycophenolate mofetil, and selenium, may be used alone or in combinations. Surgery may be required in refractory or vision-threatening cases and typically includes orbital decompression; this is particularly relevant in patients with elevated intraocular pressure or coexisting damage from glaucoma [149,150,151,152]. In this context, literature studies highlight the relevance of structural predisposition and biomechanical vulnerability in glaucoma-induced damage, regardless of intraocular pressure [153,154,155,156]. While most treatments are effective, at least to some extent, they may be associated with moderate to severe adverse effects [140]. Relatively few attempts at using SS and its analogues have been made, starting from the 1990s (Table 3).

The extraocular muscles and fat cells show immunoreactivity to IGF-1 [157], which is also implicated in the pathogenesis of pretibial myxoedema [158]. In this context, octreotide was tested in 1992 by Chang et al. [159] in six patients with Graves orbitopathy, three of whom also had pretibial myxoedema, and was found to have a therapeutic effect, while associated with minor side effects. The team of Krassas et al. [160] used Octreoscan-111 scintigraphy to predict patient response to octreotide, and then administered octreotide, concluding that it could help ameliorate thyroid eye disease, and its efficacy can be determined by a prior scintigraphy. On the other hand, four patients treated with octreotide for at least 3 months showed no significant clinical improvement in another study [161].

Using 100 μg thrice daily for 3 months, along with methimazole to maintain euthyroidism, Ozata et al. [162] found that octreotide treatment was especially effective in patients with soft tissue involvement; the mean proptosis and ophthalmopathy indices were significantly decreased at the end of therapy in 8 out of 10 patients responding to therapy.

Uysal et al. [163] tested octreotide in patients unresponsive or not suitable for glucocorticoid treatment and found it capable of improving proptosis, diplopia, and soft tissue involvement. Dickinson et al. [141], in a double-blind trial, with an initial placebo group, on the other hand, found no appreciable effects of octreotide in patients with moderate-to-severe TED. No effect against the activity of the disease was also noted by Wémeau et al. [164], who nevertheless observed a significant reduction of proptosis compared to the control group. Finally, based primarily on the patients’ Clinical Activity Score (CAS), Stan et al. [165] noted a possible improvement in values in a group with already higher CAS and an effect in cases of significant lid retraction.

Regarding other SS analogues, lanreotide was tried for the first time in 1997, by the team of Krassas et al. [166], who noted that there were significant improvements in most patients of the control group, and that lanreotide may prove more effective than other current treatments due to it being administered only once every 2 weeks. Contrary to those results, Chang & Liao [167] did not record any significant differences between the treatment and control groups, adding that, on top of the absence of any noticeable effect, the treatment itself was very costly.

Overall, there is inconsistent evidence for SS analogues in the treatment of Graves orbitopathy as the response to these agents is highly dependent on disease stage and inflammatory status; the expression of SSTRs and tracer uptake appear more closely connected to the active inflammation rather than the later fibrotic status. Therefore, early uncontrolled studies may have suggested clinical benefit, but methodologically stronger randomised clinical trials showed little or no benefit. In this context, the evidence appears weaker than that supporting the newer biological therapies available, such as teprotumumab, an IGF-1R inhibitor that directly targets a central pathogenic pathway, consolidating the current role of SS analogues as potential second-line treatment options [168,169].

**Table 3:** Clinical trials and research involving SS analogues in the management of Graves Orbitopathy (TED), arranged in chronological order.

Trial Type	Patients	Controls	Length of Trial	Dosage	Results	Year	Reference
NR	6 patients with Graves orbitopathy	0	3 m	100 μg octreotide sc thrice per day	Significant control of exopthalmos	1992	[159]
NR	12 patients with Graves orbitopathy	8 patients with Graves opthalmopathy	3 m	100 μg octreotide sc thrice per day/control group: water injections thrice per day	Significant ocular improvement in one of both eyes	1995	[160]
NR	4 patients with Graves orbitopathy	0	3 m (min.)	1 mg/d octreotide sc	No significant therapeutical benefit	1995	[161]
NR	10 patients with Graves orbitopathy	0	3 m	100 μg octreotide sc thrice per day	Improvement especially in those patients with soft-tissue involvement	1996	[162]
R	5 patients with severe Graves orbitopathy	5 patients with severe Graves orbitopathy	3 m	0.04 g lanreotide im once every 2 weeks/control group: placebo with the same adm. schedule	Statistically significant improvement in 4 patients of the control group in at least one eye	1997	[166]
NR	9 patients with Graves orbitopathy of various treatment backgrounds	0	3 m	50 μg octreotide acetate sc thrice per day	Improvement in proptosis, diplopia and soft tissue involvement	1999	[163]
R, DB	50 euthyroid patients with Graves orbitopathy (test and control groups only appliable for the 1st phase of the study)		14 m	30 mg lanreotide LAR/control group: placebo with the same adm. schedule (then both groups common treatment after 16 w)	No significant difference observed between the placebo and control groups	2004	[141]
R, DB	26 euthyroid patients with mild-to-moderate active Graves orbitopathy	25	16 w	30 mg octreotide LAR im every 4 weeks for 4 months/control group: placebo with the same adm. schedule	Effective mitigation of proptosis but not significant potency against the activity of mild disease forms	2005	[164]
R, DB	30 euthyroid patients with active Graves orbitopathy	30 euthyroid patients with active Graves orbitopathy	12 w	30 mg lanreotide im once every 2 weeks/control group: placebo treatment with the same adm. schedule	No significant different between the test and control groups	2006	[167]
R, DB	14 euthyroid patients with active Graves orbitopathy and CAS ≥ 3	11 euthyroid patients with active Graves orbitopathy and CAS ≥ 3	4 m	20 mg octreotide LAR im once per month for 4 months	Improvement in eyelid fissure width and potential improvement in CAS	2006	[165]

NR = non-randomised, R = randomised, DB = double-blinded, w = week, m = months, sc = subcutaneously, im = intramuscular, LAR = long-acting release, adm. = administration, CAS = Clinical Activity Score.

## 5. Somatostatin Analogues in the Treatment of Angiodysplasias and Gastrointestinal Bleeding

Gastrointestinal bleeding from angiodysplasias represents a therapeutic challenge, which is associated with high morbidity and mortality. GI bleeding is divided into upper and lower gastrointestinal bleeding, and it can have many causes, either frequent [170] or rare [171]. The treatment of GI bleeding is complex and usually depends on the localization and volume of the bleeding [172,173]; in spite of a number of endoscopic methods that are available, massive bleeding necessitates open surgery. Importantly, SS analogues play different roles in acute variceal bleeding, where they are a part of the first-line treatment, versus chronic or recurrent bleeding from angiodysplasias, where they are commonly employed as second-line or complementary therapies, when other treatments are not feasible or have proved ineffective.

In any case, both endoscopic and surgical interventions are often followed by recurrent bleeding [174,175,176,177]. Such occurrences necessitate pharmacological intervention, which was initially attempted with hormones, such as oestrogens and progestogens [178,179,180,181,182,183]; however, despite these positive results, other studies [184,185] indicate that such treatments, at least under certain conditions, are ineffective. Invariably, transfusions are needed when haemoglobin is less than 7 g/dL [172,173], and different medications may be given, more prominently vasoactive agents, such as SS and SS analogues [186], taking advantage of the fact that blood vessels express SSTR_2_ [119].

On the other hand, in chronic or recurrent bleeding, particularly from angiodysplasia, somatostatin and its analogues have been explored as a second-line therapeutic alternative, based on a number of case studies and trials, especially in patients not suitable for endoscopic or surgical interventions (Table 4).

The first results of the potential usefulness of SS in bleeding of the GI tract were demonstrated in research efforts during 1980 [187,188], following experiments performed in rats [189,190] and humans [191]. A randomised double-blind clinical trial performed in 1985, in patients with massive upper GI bleeding, found that while, compared to the placebo group, SS did not reduce mortality rate or the need for blood transfusions significantly, it did reduce the number of patients requiring surgery [192]. An earlier trial by Kravetz et al. [193] in 1984, comparing SS and vasopressin, found them to be of similar effectiveness, but SS had a lower rate of complications.

The first ever multicentre trial on the effectiveness of SS in GI bleeding was performed by Torres et al. [194] and found it superior to a combination of cimetidine and pirenzepine. In the same year, another pilot study found that octreotide stopped bleeding and prevented rebleeding in 80% of participants, in cases of severe acute upper GI bleeding due to peptic ulcers, with no side effects being reported [195]. SS was compared to ranitidine and to placebo, in the case of bleeding due to acute ulcer disease, by Coraggio et al. [196] in 1989, who determined it to be more effective than both, especially in the case of severe and moderate haemorrhages. Contrary to these results, in an earlier double-blind trial, neither ranitidine nor SS was found to have any appreciable effect in upper GI haemorrhage [197]. Compared to secretin, SS was found to be more efficient in controlling active bleeding, although mortality and rebleeding did not differ significantly between the two groups [198]. Previously, SS had not been found effective in managing the bleeding of oesophageal varices, although there were some limitations acknowledged by the authors in this particular study [199].

Research into the management of angiodysplasias with octreotide started in the 1990s [200]. A randomised controlled trial the next year demonstrated that, compared to vasopressin, octreotide was more effective in controlling the bleeding at its initial stages and had fewer side effects [201]. Bleeding prevention and control were achieved by Rossini et al. [202] in three patients, with a minimum octreotide dose of 0.1 mg sc twice a day. A prospective trial by Lin et al. [203], comparing octreotide to ranitidine and to heater probe thermocoagulation, determined that the invasive approach offered better therapeutic outcomes. At this point, octreotide, when being used along with variceal ligation, has been found to be able to reduce further rebleeding episodes [204] and control variceal bleeding if used in place of emergency sclerotherapy [205].

A single case study, with SS being used instead of octreotide, was reported by Andersen & Aaseby [206]. In the same year, a large randomised controlled trial compared SS to terlipressin and found that both treatment schemes had about 80% effectiveness, with the possibility of being maintained for prolonged periods to prevent rebleeding. Side effects were significantly higher in the terlipressin group, however [207]. No significant side effects for either octreotide or SS were noted in the successful trial of Kouroumalis et al. [208], in patients where the bleeding was attributable to portal hypertensive gastropathy. One year earlier, octreotide had not been found to be superior to endoscopic therapy in patients with upper GI variceal bleeding [209]. On the other hand, a continuous SS infusion was found to be as effective as endoscopic variceal ligation, and to have a lower rate of complications [210].

A few years later, in 1999, Nardone et al. [211] treated 17 patients with various degrees of angiodysplasia and some with “watermelon stomach”; of them, 10 patients had complete bleeding cessation, and it was concluded that octreotide is a safe and beneficial therapy, especially for those patients who cannot undergo surgery for any reason. The term “watermelon stomach” was introduced in the 1980s [212] to describe gastric antral vascular ectasia (GAVE) [213]. Two cases where GIT angiodysplasia was associated with von Willebrand’s disease (VWD), where all other treatment measures had failed, were presented by Bowers et al. [214] in 2000.

In 2000, Junquera et al. [215] compared administration of SS alone, compared to SS and isosorbide-5-mononitrate, in patients with GI bleeding in the context of liver cirrhosis. SS alone was found to be more effective and associated with fewer adverse reactions. In the same year, Zuberi & Baloch [216] published the results of a trial on patients with variceal bleeding in the context of low-risk liver cirrhosis, where they compared the results of endoscopic variceal sclerotherapy alone and in combination with octreotide administration over 5 days. They noted that the bleeding arrest results in both groups were similar, but the need for blood transfusions and for prolonged hospitalization was reduced in the octreotide group.

In one anticoagulated patient, due to prior cardiac surgery, with GIT bleeding due to an angiodysplasia, which could not be visualized in endoscopic exams, a 5-day continuous octreotide drip was enough to keep the patient stable for 5 months post-discharge [217]. In a similar case, in an elderly patient, where the cause of coagulopathy was Glanzmann thrombasthenia, octreotide was used in association with other drugs, and helped to manage bleeding and reduce the need for transfusions [218].

LA octreotide was found to reduce hospital admission and anaemia occurrences, with few adverse effects, in three patients with recurrent gastrointestinal bleeding due to angiodysplasia [219]. It was concluded that LA octreotide could be a safe alternative for patients not eligible for surgical or endoscopic therapy [219].

The randomised clinical trial conducted by Junquera et al. [220] observed that while the placebo and octreotide treatment groups had similar outcomes, octreotide treatment was possibly effective in preventing rebleeding. When octreotide or terlipressin was used as an adjuvant treatment, along with endoscopic band ligation, there seemed not to be any difference in outcome between the two groups [221]. In a rather different trial, both octreotide and SS were tried in cirrhotic patients with variceal treatment, after endoscopic therapy, and it was found that only could increase the hepatic vein pressure gradient, thus reducing the odds of treatment failure [222].

In the trial of Seo et al. [223], patients with variceal GI bleeding, in the context of liver cirrhosis, were randomised in three groups, receiving terlipressin, somatostatin, or octreotide. The results confirmed the usefulness of the used drugs as adjuvant treatments to endoscopic interventions, but did not discover any notable differences in effectiveness between them.

In 2023, in cirrhotic patients with upper GI bleeding, Zhu et al. [224] found that octreotide was superior to putuitrin, with advantages of quick onset, short haemostasis time, and fewer adverse reactions. The most recent trial involving octreotide was the OCEAN study, whose protocol had been proposed in 2016 [225]. This was a multicentre randomised controlled trial, where it was determined that octreotide effectively reduces transfusion requirements and the need for endoscopic therapy in patients where anaemia is caused by GIT bleeding [226].

Long-term lanreotide was tried in patients with occult gastrointestinal bleeding or gastrointestinal angiodysplasia, who were refractory or not suitable for other therapies. After a minimum of 6 months of therapy and a 3-year follow-up, some patients achieved a complete response to therapy, and the majority had a positive response to therapy [227].

Regarding other SS analogues, very few experimental and clinical data are available. There was another trial in a French hospital, by Calès et al. [228], in 2001, for the purposes of haemostasis, combined with endoscopic intervention, in patients suffering from variceal bleeding in the context of liver cirrhosis; results in short-term mortality were similar between the vapreotide and the placebo group, even though haemostasis seemed to be more efficient in the test group. However, the real efficacy of vapreotide, based on the results of this trial, is rather controversial [229].

Finally, the efficacy of pasireotide LAR in cases of recurrent GI bleeding due to dysplasias was demonstrated in the phase II ANGIOPAS multicentre study, where the transfusion requirements of patients in the test group were reduced compared to those of patients in the control group. Glycaemic control was impaired in very few patients [230].

Therefore, somatostatin and its analogues are well-established in the management of acute variceal bleeding and are candidates for second-line treatment in non-variceal chronic bleeding, in selected or refractory cases.

**Table 4:** Clinical trials and research involving SS and its analogues in the treatment of angiodysplasias and GI bleeding, arranged in chronological order.

Trial Type	Patients	Controls	Length of Trial	Dosage	Result	Year	Reference
R	10 patients with peptic ulcer haemorrhage unsuitable for surgery	10 patients with peptic ulcer haemorrhage unsuitable for surgery	2.5 y	250 μg/h continuous SS IV infusion for 48–120 h, after IV bolus adm. of 250 μg by an infusion pump/control group: 200 μg cimetidine IV every 4 h for 48–120 h.	SS is a safe conservative treatment in persistent peptic-ulcer bleeding and much more effective compared to cimetidine	1980	[187]
R, B	30 patients with variceal bleeding	31 patients with variceal bleeding	48 h	250 μ grams/h continuous SS IV infusion after a bolus of 50 μ grams IV bolus/control group: 0.4 U/min continuous vasopressin IV infusion	SS is as effective as vasopressin in controlling variceal haemorrhage but has a much lower complication rate	1984	[193]
R, DB	46 patients with massive upper GI bleeding	49 patients with massive upper GI bleeding	28 m	250 μg/h continuous SS IV infusion, after an initial bolus of 250 μg IV/control group: placebo treatment	Reduction of patients requiring surgery compared to the placebo group	1985	[192]
R, DB	30 patients with upper GI bleeding (not due to oesophageal varices)	30 patients with upper GI bleeding (not due to oesophageal varices)	varied	250 μg/h continuous SS IV infusion and placebo of cimetidine and pirenzepine/control group: 200 mg cimetidine IV every 4 h & 10 mg pirenzepine IV every 8 h. plus placebo SS administration	In 90% of patients in the SS group bleeding stopped, compared to about 67% in the cimetidine group	1986	[194]
NR	10 patients with acute severe upper GI bleeding due to peptic ulceration	0	4 d	100 μg continuous SS IV infusion at the beginning of treatment (for 1 h) and then 25 μg/h continuous IV infusion over 72 h	Cessation of bleeding and prevention of rebleeding in 80% of patients with no side effects being reported	1986	[195]
R, DB	96 patients with massive GI bleeding divided into 3 groups (31:33:32 patients)		120 h	250 μg/h continuous SS IV infusion (1st group); 300 mg/d ranitidine continuous IV infusion (2nd group)/placebo (3rd group)	Both ranitidine and SS did not seem to alter the clinical course of patients	1986	[197]
NR	220 patients classified into severe, moderate and mild haemorrhage groups (each group divided into 3 subgroups)		72 h	250 μg/h continuous SS IV infusion (1st subgroup); 50 mg ranitidine IV every 4 h (2nd subgroup); placebo (3rd subgroup)	SS was found to be more effective than ranitidine, which was in turn more effective than placebo	1989	[196]
R, DB	48 patients with actively bleeding oesophageal varices	36 patients with actively bleeding oesophageal varices	30 h	250 μg rapid SS IV infusion in physiological saline solution and concomitant 250 μg/h continuous SS IV infusion in sodium chloride solution every 12 h/control group: placebo treatment in a similar administration protocol	SS did not seem to be effective in the management of oesophageal varices	1989	[199]
R, DB	31 patients with actively bleeding gastric erosions	32 patients with actively bleeding gastric erosions	72 h (max.)	Initial endoscopy and afterwards with 250 μg SS bolus IV followed by a continuous IV infusion at a rate of 250 μg/48 h; control group: initial endoscopy and afterwards continuous secretin infusion at a rate of 0.5 clin. U/kg/h	SS administration resulted in better bleeding control, reduction of the need for transfusions and surgical intervention	1992	[198]
R	24 cirrhotic patients with variceal bleeding	24 cirrhotic patients with variceal bleeding	24 h	100 μg initial bolus of octreotide IV and then 25 μg/h IV for 24 h/control group: 0.4 U/min vasopressin for 24 h	No difference in mortality between two groups, but significantly less side effects and better control of initial variceal bleeding in the octreotide group	1992	[201]
R	49 patients with acute variceal haemorrhage	49 patients with acute variceal haemorrhage	48 h	50 μg octreotide IV bolus at the start of treatment and 50 μg/h continuous IV infusion/control group: emergency sclerotherapy	Octreotide is equally as effective as emergency sclerotherapy in controlling variceal haemorrhage	1993	[205]
NR	3 patients with prior bleeding due to small bowel angiodysplasia	0	10–40 m	0.1 mg octreotide sc twice per day	Octreotide is efficient in the control and prevention of bleeding due to diffuse small bowel angiodysplasia	1993	[202]
R	54 patients with actively bleeding peptic ulcers or non-bleeding visible vessels at the ulcer base divided into three groups (19:20:15)		72 h	100 μg octreotide IV bolus at the beginning and then 25 μg/h continuous IV infusion (1st group); heater probe thermocoagulation (2nd group); 100 mg ranitidine IV every 12 h (3rd group)	Heater probe thermocoagulation was more effective than octreotide in controlling upper GI bleeding	1995	[203]
R	47 patients with oesophageal varices with active bleeding or recent haemorrhage	47 patients with oesophageal varices with active bleeding or recent haemorrhage	5 d	50 μg octreotide IV bolus during endoscopy and then 50 μg/h continuous infusion, in addition to variceal ligation/control group: variceal ligation	The use of octreotide along with variceal ligation significantly reduces rebleeding episodes	1995	[204]
NR	1 patient with recurrent anaemia due to angiodysplasia	0	26 m	100 μg SS sc twice per day	Significant decrease of GIT bleeding	1996	[206]
R, DB	81 patients with variceal bleeding	80 patients with variceal bleeding	48 h (max.)	250 μg continuous SS IV infusion, after an initial bolus of 250 μg (max. 3 add. boluses allowed)/control: 2 mg terlipressin IV every 4 h	Both treatment schemes were found to be equally effective (at about 80%) and well-tolerated for prolonged treatment periods	1996	[207]
R	73 patients with upper GI variceal bleeding	77 patients with upper GI variceal bleeding	48 h	50 μg/h octreotide IV infusion for 28 h/control group: emergency sclerotherapy	The results in the octreotide and sclerotherapy groups were similar	1997	[209]
R	99 patients with acute variceal haemorrhage	79 patients with acute variceal haemorrhage	5 d (max.)	250 μg/h continuous SS IV infusion after an initial 250 μg injection every 24 h (+possibility for 2 additional SS boluses upon haemorrhage reactivation)/control group: endoscopic injection sclerotherapy	Continuous SS infusion is as effective as sclerotherapy in preventing rebleeding and reducing mortality, but has a lower rate of complications	1998	[210]
NR	11 patients with acute GI bleeding from portal hypertensive gastropathy	15 patients with acute GI bleeding from portal hypertensive gastropathy	3 d	250 μg SS IV bolus initially followed by 250 μg/h continuous IV infusion for 3 d/control: 100 mg octreotide IV bolus initially followed by 50 μg/h continuous IV infusion for 3 d	SS represents a safe and effective treatment for acute severe bleeding from portal hypertensive gastropathy	1998	[208]
NR	17 patients with different degrees of GIT angiodysplasia-induced bleeding or watermelon stomach	0	6 m	0.1 mg octreotide sc 3 times per day	Significant control of GIT in most patients	1999	[211]
R, DB	30 cirrhotic patients with GI variceal bleeding	30 cirrhotic patients with GI variceal bleeding	72 h	250 μg SS IV bolus initially followed by 250 μg/h continuous IV infusion plus 40 mg/12 h isosorbide-5-mononitrate orally/control: same scheme with placebo instead of isosorbide-5-mononitrate	The administration of isosorbide 5-mononitrate to patients already treated with SS does not improve therapeutical efficiency and increases the incidence of adverse effects	2000	[215]
NR	1 patient with VWD type 1	0	13 m	500 μg octreotide IV for 2 successive doses and then 250 μg sc, reduced to 100 μg sc	Significant decrease of GIT bleeding, with tolerable adverse effects	2000	[214]
NR	1 patient with VWD type 2A	0	~4 m	500 μg octreotide IV for 2 successive doses and then 300 μg sc twice daily, reduced t 250 μg sc	Significant control of GIT with steatorrhea as side effect		
R, DB	35 patients with variceal bleeding in the context of low-risk liver cirrhosis	35 patients with variceal bleeding in the context of low-risk liver cirrhosis	5 d	Endoscopic variceal sclerotherapy and 50 μg/h octreotide continuous IV infusion for 5 d/control group: Endoscopic variceal sclerotherapy with 3–5 mL ethanolamine oleate per varix and placebo injection at 50 μg/h	While bleeding arrest did not differ significantly between the two groups, rebleeding episodes, hospital stay and then need for blood transfusions were reduced in the octreotide group	2000	[216]
NR	3 patients with recurrent GIT bleeding due to angiodysplasia	0	15–17 m	20 mg octreotide LAR im once per month	Significant control of GIT bleeding in patients of old age and/or concomitant disorders	2001	[219]
DB	111 patients with variceal bleeding due to liver cirrhosis	116 patients with variceal bleeding due to liver cirrhosis	47 d	50 μg vapreotide IV bolus followed by continuous infusion of 50 μg/h for 5 d	Vapreotide may be useful but it did not significantly affect short-term outcomes	2001	[228]
NR	1 anticoagulated patient with GIT bleeding due to diffuse angiodysplasia	0	5 m	Continuous octreotide drip of 50 μg/h for 5 d	Significant control of GIT bleeding and elevation of Hb levels	2002	[217]
R	32 patients with GI bleeding due to angiodysplasias	38 patients with GI bleeding due to angiodysplasias	1–2 y	50 μg/12 h octreotide sc for 1–2 y/control group: placebo treatment	Octreotide treatment is possibly beneficial in preventing rebleeding episodes	2007	[220]
R, DB	16 cirrhotic patients with bleeding varices	17 cirrhotic patients with bleeding varices	120 h	50 μg/h octreotide continuous IV infusion (1st group)/(2nd group) 250 μg/h continuous SS IV infusion	SS prevents the post-endoscopic increase in hepatic venous pressure gradient	2007	[222]
R, DB	161 cirrhotic patients with oesophageal variceal bleeding	163 cirrhotic patients with oesophageal variceal bleeding	72 h	100 mL bolus of 100 μg octreotide IV octreotide and a placebo 10 mL IV bolus as placebo for terlipressin; then 50 μg/h octreotide continuous IV infusion for 72 h and a six hourly IV injection of 5 mL of placebo of terlipressin equivalent in amount of 1 mg terlipressin for 72 h/control group: 2 mg (10 mL) terlipressin IV bolus followed by 1 mg (5 mL) IV every 6 h along with a placebo bolus of 100 mL as placebo for octreotide (along with endoscopic intervention at both groups)	Octreotide was not found to alter the clinical outcome significantly compared to terlipressin when both drugs were used adjuvant to endoscopic variceal band ligation	2009	[221]
R	780 patients with variceal GI bleeding (261:259:260)		5 d	2 mg terlipressin IV bolus at the start of treatment, followed by 1 mg IV/6 h; 250 μg SS IV bolus at the start of treatment, followed by 250 μg/h continuous IV infusion; 50 μg octreotide IV bolus at the start of treatment followed by 25 μg/h continuous IV infusion	Similar effectiveness of all drugs as adjuvant therapies to endoscopic interventions	2014	[223]
R, DB (ANGIOPAS study)	10 patients with ≥6 blood units transfusion requirement	12 patients with ≥6 blood units transfusion requirement	6 m	60 mg pasireotide LAR im every 28 d/control group: placebo	Transfusion requirements in patients with recurrent bleeding due to GI dysplasias was reduced	2018	[230]
NR	27 patients of different treatment backgrounds	0	≥6 m	60 mg or 90 mg lanreotide sc mg every 28 d	Improvement of anaemia and reduced healthcare costs	2018	[227]
R, B	66 cirrhotic patients with upper GI bleeding	66 cirrhotic patients with upper GI bleeding	24–72 h	0.1 mg octreotide acetate + 20 mL sodium chloride (0.9)% IV, then 500 mL sodium chloride or glucose + 0.5 mg octreotide continuous IV administration at 30–50 μg/h/control group: 6 units pituitrin IV, then 500 mL glucose (5%) + 36 μ pituitrin IV	Octreotide is superior to pituitrin and is better tolerated by the patients	2023	[224]
R, B (OCEAN study)	31 patients with transfusion-dependent anaemia due to GI angiodysplasia	31 patients with transfusion-dependent anaemia due to GI angiodysplasia	1 y	40 mg octreotide LAR im every 28 d/control group: standard of care	Octreotide reduces the mean number of blood transfusion units required and the need for endoscopic intervention	2024	[226]

NR = non-randomised, R = randomised, DB = double-blinded, B = blinded, h = hours, d = days, m = months, sc = subcutaneously, im = intramuscular, IV = intravenous, SS = somatostatin, GIT = gastrointestinal, LAR = long-acting release.

## 6. SS and Its Analogues in the Treatment of Acute Pancreatitis

In general, in the treatment of acute pancreatitis, the first line of treatment or management is pharmacological, with minimally invasive measures being the second-line choice, and then surgery being the last option [231]. Except for urgent intervention in specific clinical cases, such as the performance of ERCP-induced pancreatitis and cholangitis or in acute gallstone pancreatitis with common bile duct obstruction, it is generally recommended to delay any invasive approach. No definitive pharmacological treatment of acute pancreatitis has been found, and antibiotics and pain medication are given as needed; the main goal of treatment is to treat the cause and prevent recurrence.

Initial experiments on the effects of SS in acute pancreatitis were studied in a dog model [232]; previous experiments had indicated that SS can effectively inhibit cholecystokinin (CCK) release from the pancreas [233]. As the secretory volume in the duodenum had previously been found to be reduced following SS administration by Lankisch et al. [234], the next step was to try out SS and its analogues in acute pancreatitis patients (Table 5). The results of the trial of Raptis et al. [235] on healthy volunteers suggested that SS could have a potential role in the treatment of acute pancreatitis.

In the early to late 1980s, a number of researchers first used SS in the management of acute pancreatitis, mostly in Italy [236,237,238,239,240,241]. Of note was the APTS (acute pancreatitis with somatostatin) study, involving 19 medical centres from different countries, including only patients with severe acute pancreatitis. Patients were chosen based on a combination of symptoms and blood test values [232].

In 1989, a prospective randomised study was conducted to assess the effects of SS in acute pancreatitis. Blood test results were better in the study group—white blood cell (WBC) count, lactate dehydrogenase (LDH), and urea levels were lower—and there was a reduction in complications [242]. The results of this trial were supported by the similar results of D’Amico et al. [241]. On the other hand, the randomised double-blind trial of Gjørup et al. [243] did not observe significant differences between the control group, treated with a continuous SS infusion, and the placebo group. Two years later, in 1994, Luengo et al. [244] found that SS administration was sufficient to cause a reduction in pancreatic lesion evolution and in the length of hospital stay. In another clinical trial, it was found that SS administration was able to reduce patient mortality but not symptom severity [245], while the clinical trial of Planas et al. [246] found SS administration to have a very slight positive effect, being only able to somewhat reduce the need for surgery due to local complications. An interesting study performed in 2013 by Wang et al. [247] discovered that the combination of somatostatin, ulistatin, and *Salvia miltiorrhiza* reduced the incidence of pancreatic sepsis, multiple organ dysfunction, and mortality rates. The levels of TNF-a and IL-6 were also found to be reduced compared to the control group.

The first significant evidence on the effectiveness of octreotide in acute pancreatitis came from the 1993 study of Beechey-Newman [248], who determined that octreotide treatment, in addition to conventional treatment, was sufficient to reduce the indicators of poor prognosis in the first 48 h after admission. A rather different study took place the next year, where it was tested whether octreotide administration would be effective in protecting patients against endoscopic sphincterotomy (EST)-induced acute pancreatitis; no difference in acute pancreatitis incidence between the control and the test groups was found [249]. Similarly, McKay et al. [250] found that there were no significant differences in the outcomes between the placebo and octreotide groups. In another study, this time in patients about to undergo diagnostic and therapeutic endoscopic retrograde cholangiopancreatography (ERCP), octreotide was again found incapable of reducing the incidence of post-ERCP acute pancreatitis and the rise of serum amylase levels [251]. Some other trials on patients about to undergo EST or ERCP have been performed, such as the one by Baldazzi et al. [252], but not all the results are available [253]. Few studies reported encouraging results in that respect, such as the study of Thomopoulos et al. [254].

Meanwhile, in 1995, Paran et al. [255] found octreotide to be superior to the conservative treatment administered to the control group, results that were in accordance with the later trial of Karakoyunlar et al. [256]; in patients with acute pancreatitis, after 14 days of treatment, octreotide was found to be potentially effective by Paran [257]. The larger multicentre trial of Uhl et al. [258] showed, on the other hand, that there was no difference between groups treated with different octreotide doses and placebo treatment. In a large study on obese patients with acute pancreatitis, octreotide administration was found to be able to prevent progression to its severe form by normalizing plasma SS levels and reducing circulatory cytokines [259].

Finally, the only significant study involving lanreotide was performed in 2004 by Lévy et al. [260], who demonstrated that one intramuscular injection of lanreotide 30 mg on the day before refeeding may reduce the pain relapse in patients with acute necrotizing pancreatitis; these encouraging results warrant a phase III study for confirmation.

**Table 5:** Clinical trials and research involving SS analogues in the treatment of acute pancreatitis, arranged in chronological order.

Trial Type	Patients	Controls	Length of Trial	Dosage	Result	Year	Reference
R	35 patients admitted for acute pancreatitis	36 patients admitted for acute pancreatitis	5 d	250 μg/h of intravenous bolus of SS every hour, then titrated to 100 μg per hour for 48 h in addition to conservative treatment/control: only conservative regimen comprising cephoperazone	Improvement in biochemical markers and reduction of complications	1989	[242]
R, DB	33 patients with acute pancreatitis	30 patients with acute pancreatitis	72 h	250 μg/h of intravenous bolus of SS as a continuous infusion per hour/control: placebo treatment	Slightly faster decrease of serum amylase but otherwise no significant difference to the control group	1992	[243]
NR	9 patients with acute pancreatitis	10 patients with acute pancreatitis	48 h	Conventional therapy plus 250 μg octreotide sc followed by 0.5 μg/kg/h IV, 1 h after the initial administration/control: conventional therapy	Reduction in the frequency of indicators for poor prognosis	1993	[248]
R	75 patients scheduled for EST	76 patients scheduled for EST	48 h	0.1 mg of octreotide acetate sc, 120 min and then 30 min pre-intervention and 4 h post-intervention/control: placebo treatment at the same time intervals	Octreotide administration was not enough to prevent post-EST acute pancreatitis	1994	[249]
R	50 patients scheduled for ERCP or EST	50 patients scheduled for ERCP or EST	~24 h	0.1 mg of octreotide acetate sc, 45 min pre-intervention and 6 h post-intervention/control: placebo treatment at the same time intervals	n/a	1994	[252]
R	50 patients with acute pancreatitis	50 patients with acute pancreatitis	48 h	250 μg of intravenous bolus of SS at the beginning of therapy and thereafter a continuous infusion of 250 μg/h/control: no therapy	The evolution of the pancreatic lesions was slower and the length of hospitalisation shorter in the test group	1994	[244]
R	19 patients with acute pancreatitis	19 patients with acute pancreatitis	various	0.1 mg octreotide injected sc three times per day/control: conservative treatment	The complication rate, death rate and hospital stay were lower in the treatment group	1995	[255]
R, DB	28 patients with moderate to severe acute pancreatitis	30 patients with moderate to severe acute pancreatitis	5 d	40 μg/h continuous octreotide IV infusion/control: placebo with the same administration method	No significant difference in the incidence of complications or mortality between the test and control groups	1997	[250]
R, DB	47 total patients with acute pancreatitis from different causes (test and control group no n/a)		48 h	250 μg/h of intravenous bolus of SS as a continuous infusion/control: placebo treatment	Severity was similar between both groups, but mortality was significantly reduced in the test group	1997	[245]
R	24 patients with acute pancreatitis	22 patients with acute pancreatitis	10 d	Conventional treatment along with an initial IV SS bolus of 3.5 μg/kg, followed by a continuous IV perfusion of 3.5 μg/kg per h/control: only conventional treatment	Slight reduction in need for surgery due to local complication; otherwise, no significant differences between groups	1998	[246]
R	37 patients about to undergo ERCP	36 patients about to undergo ERCP	~24 h	0.1 mg octreotide sc 30 min before and 6 and 16 h post-ERCP/control: placebo treatment	No statistically significant difference between groups in terms of acute pancreatitis incidence and serum amylase levels	1998	[251]
R	22 patients with acute pancreatitis	21 patients with acute pancreatitis	48 h	0.5 μg/kg/h octreotide in continuous IV infusion/control: placebo treatment	Beneficial effects of high-dose octreotide regarding serum amylase levels, pancreatic oedema and return to oral feeding	1999	[256]
R, DB	98 + 101 patients with acute pancreatitis	103 patients with acute pancreatitis	7 d	100 and 200 μg of octreotide sc 3 times/day (for treatment groups 1 & 2 respectively)/control: placebo treatment	No significant differences in any markers between groups	1999	[258]
R, B	25 patients with acute pancreatitis	25 with acute pancreatitis	14 d	0.1 mg octreotide sc three times per day (every 8 h)/control: conservative treatment	Potential beneficial effect of octreotide	2000	[257]
NR	23 patients with acute necrotizing pancreatitis	0	5 d	30 mg lanreotide im once the day before refeeding	Decrease of pain after refeeding	2004	[260]
R, DB	100 patients scheduled for ERCP	100 patients scheduled for ERCP	~24 h	500 μg octreotide sc 3 times per day 24 h before the ERCP procedure	24 h prophylaxis with octreotide is effective in preventing post-ERCP pancreatitis	2006	[254]
R	79 obese patients with acute pancreatitis	82 patients with acute pancreatitis	72 h	50 μg/h octreotide continuous IV infusion & conventional treatment/control: conventional treatment	Octreotide treatment was found able to prevent progression to severe acute pancreatitis	2012	[259]
R, DB	306 patients with acute pancreatitis (randomly divided into 5 groups)		10 d	5 treatment groups: SS, SS + ulinastatin, SS + *S. miltiorrhiza*, SS + ulinastatin + *S. miltiorrhiza*, basic treatment	All groups with the exception of the basic treatment group had decreased rates of pancreatic sepsis and multiple organ dysfunction	2013	[247]

NR = non-randomised, R = randomised, DB = double-blinded, B = blinded, h = hours, d = days, sc = subcutaneously, im = intramuscular, IV = intravenous, SS = somatostatin, EST = endoscopic sphincterectomy, ERCP = endoscopic retrograde cholangiopancreatography.

## 7. SS Analogues in the Management of Dumping Syndrome

Dumping syndrome was first described in 1922 [261], and occurs due to the rapid movement of hyperosmolar chyme from the stomach into the small intestine; it is a common complication of oesophageal, gastric, and bariatric surgeries [262]. It can be divided into early dumping syndrome—due to overly rapid nutrient delivery in the small intestine—and late dumping syndrome, which essentially is reactive hypoglycaemia, and manifests accordingly. Crucially, patients are symptom-free when fasting, and this can be a useful distinguishing feature during differential diagnosis [262]. Usually, dumping syndrome is preventable or treatable by diet alone, and only about 1% of patients will end up developing relevant symptoms [263]. The severity of dumping syndrome predominantly depends on the extent and type of GI surgery [264,265]. Administration of viscosity-modifying agents or acarbose for early and late dumping syndrome, respectively, is the next step, if dietary interventions fail, followed by SS analogue administration [262]; their use is based on the reduction of intestinal motility they induce, and, as such, they have found a number of applications in this particular pathology (Table 6).

Initial research into the potential use of SS in the management of dumping syndrome was performed by Mörz et al. [266] in 1982, followed by that of Reasbeck et al. [267], based on the principle that SS would suppress excess gastric secretions. Indeed, there was cessation of diarrhoea, but also abdominal pain in three out of four subjects in dumping provocation tests. Very good results in both early and late dumping syndrome were recorded in the subsequent trial of Hopman et al. [268], who used octreotide instead of SS; one year later, in 1989, Tulassay et al. [269], using octreotide 15 min prior to an oral glucose tolerance test (OGTT) in patients having undergone Billroth II gastric resection, determined that it could alleviate the symptoms of early and late postprandial dumping syndromes. In 10 patients with severe postgastrectomy dumping syndrome, Geer et al. [270] noted a marked improvement in both symptoms and biochemical markers, as did Richards et al. [271], who also noted that octreotide acetate altered GI motility from a postprandial to a fasting pattern.

In 2005, an open study was performed by Penning et al. [272] over a period of 7 months, where octreotide was effective in improving the quality of life and body weight in patients with severe dumping syndrome. In addition, it was demonstrated that octreotide LAR was more effective than octreotide. Similar results were recorded by Arts et al. [273] in 2009.

The first use of pasireotide in patients with dumping syndrome was in 2014, when the team of Deloose et al. [274], in a double-blind pilot study, demonstrated that pasireotide exhibited potent pharmacodynamic effects on relevant pathophysiological mechanisms in dumping syndrome. Following this, a phase II, multicentre study was organized in four phases, lasting for 6 or 12 months, depending on whether the patients elected to join the extension phase [275]. While the number of patients studied was relatively small, 43 and 23 patients for the original phase and the extension, respectively, it still remains amongst the largest studies performed on dumping syndrome to date. Further studies, with a greater duration, and possibly assessing other parameters apart from OGTT response, are required to fully document the efficacy of pasireotide. Nevertheless, it was clearly demonstrated that pasireotide reduced the incidence of hypoglycaemia and improved the symptoms of both early and late dumping syndrome [275]. Finally, one phase II study used lanreotide, succeeding in treating most of the early dumping syndrome symptoms, albeit with very little improvement in the patients’ quality of life due to the drug’s side effects [276].

**Table 6:** Clinical trials and research involving SS analogues in the management of dumping syndrome, arranged in chronological order.

Trial Type	Patients	Controls	Length of Trial	Dosage	Results	Year	References
R, DB, CO	4 patients with dumping syndrome after gastric surgery	4 patients with dumping syndrome after gastric surgery	n/a	250 μg SS IV bolus followed by 300 μg/h continuous IV infusion/control group: placebo treatment	Suppression of diarrhoea but pain on dumping provocation tests	1986	[267]
R, DB, CO	6 patients with gastrointestinal surgery (different types)	6 patients with gastrointestinal surgery (different types)	24 h	50 μg octreotide sc prior to meal ingestion/control group: placebo treatment prior to meal ingestion	Dramatic improvement of postprandial dumping symptoms or reactive hypoglycaemia (in patients with late dumping symptoms)	1988	[268]
R, DB, CO	8 patients with Billroth II gastric resection	8 patients with Billroth II gastric resection	24 h	50 μg octreotide sc 15 min before oral glucose challenge/control group: placebo treatment 15 min before oral glucose challenge	Prevention of hypoglycaemia and stable plasma insulin and GIP concentrations in the test group	1989	[269]
R, DB, CO	10 patients with severe postgastrectomy dumping syndrome	10 patients with severe postgastrectomy dumping syndrome	48 h	100 μg octreotide acetate sc 30 min prior to test meal/control group: placebo treatment 30 min prior to test meal	Both the symptoms of dumping syndrome and the associated biochemical markers showed marked improvement	1990	[270]
R, DB, CO	6 patients with severe early dumping syndrome	6 patients with severe early dumping syndrome	48 h	100 μg octreotide acetate sc 20 min prior to test meal	Remission of symptoms associated with dumping syndrome	1990	[271]
NR	12 patients with severe dumping syndrome	0	7 m	Octreotide sc once per day for 2 w and then 10–20 mg octreotide LAR sc at different time intervals (4 doses in total)	Increase in body weight and improvement in patient quality-of-life	2005	[272]
NR	30 patients with post-operative dumping syndrome	0	3 m	0.5 mg octreotide sc 3 times/d for 3 d and then 20 mg octreotide LAR sc once per month for 3 months	Improvement in OGTT, dumping severity score and quality-of-life	2009	[273]
R, DB, CO	9 patients with postoperative dumping syndrome (score > 10 on a dumping syndrome severity scale)	9 patients with postoperative dumping syndrome (score > 10 on a dumping syndrome severity scale)	5 w	300 μg pasireotide sc 3 times/day for 2 w/control group: placebo treatment with same regimen (1 week washout period between treatment change)	Pasireotide favourably alters the pathophysiological features of both early and late dumping syndrome	2014	[274]
NR	43 patients with late dumping syndrome (initial phase)/23 patients with late dumping syndrome (extension phase)	0	6/12 m	Pasireotide sc (3 times/d for 3 m) and pasireotide im (once per month for 3 m) and (optional extension:) octreotide im once per month for 9 m (different doses depending on the stage)	Pasireotide successfully resolved the symptoms of both early and late dumping syndrome	2018	[275]
R, DB, CO	24 patients with postoperative dumping syndrome (score > 10 on a dumping syndrome severity scale)	24 patients with postoperative dumping syndrome (score > 10 on a dumping syndrome severity scale)	30 w	90 mg lanreotide sc once per month for 3 months/control group: placebo treatment with same regimen (washout period of 8 w in-between)	Successful treatment of early dumping syndrome symptoms but not significant quality-of-life improvement	2019	[276]

CO = crossover, NR = non-randomised, R = randomised, DB = double-blinded, h = hours, d = days, w = weeks, m = months, sc = subcutaneously, im = intramuscular, IV = intravenous, SS = somatostatin, GIP = gastric inhibitory peptide, LAR = long-acting release.

## 8. Discussion

From the information presented herein, it can be concluded that the three somatostatin analogues used in current clinical practice, octreotide, lanreotide, and pasireotide, can be of use in different pathologies, as either monotherapies or in association with other drugs and interventions, with SS itself being a viable treatment option in a handful of specific clinical settings.

Octreotide has been used in the treatment of thymic tumours, neuroendocrine tumours (NETs), Merkel-cell carcinoma, and HCC. Lanreotide has also been used in the treatment of NETs and HCC; SS analogues have also been used in the treatment of hypoglycaemia due to nesidioblastosis since the 1980s [277,278]. Fairly recently, a case of a patient with hypoglycaemic episodes due to nesidioblastosis was treated with pasireotide. This was the first time that adult nesidioblastosis was thus treated, and the patient remained free of hypoglycaemic episodes during a follow-up of three years [279]. Aside from neoplastic pathologies, SS analogues represent effective treatment options for patients with congenital hyperinsulinism, GI bleeding due to angiodysplasias or other causes, acute pancreatitis, dumping syndrome, and specific ocular pathologies associated with hyperthyroidism (Table 7 and Table 8).

**Table 7:** First-line treatments and basic information on SS and SS analogue use for the pathologies referred to within the text. The references correspond to SS and SS analogues as described in the relevant tables; for the first-line treatments and their adverse effects and limitations, the references are those provided in the relevant text passages.

Pathology	First-Line Treatment	Adverse Effects	SS Analogues	Doses		Results	References
				Min	Max		
Congenital hyperinsulinism	Pancreatectomy for focal lesions; medical therapy with diazoxide (+/−IV dextrose and glucagon) for diffuse hyperinsulinism	Impaired glucose metabolism after surgery; increased risk for developing diabetes; rare cases of necrolytic migratory erythema skin rash after glucagon administration	Octreotide	5 μg/kg/d continuous sc infusion	30 μg/kg/d continuous sc infusion	Octreotide treatment is equally or as effective as first-line non-surgical therapies and is usually better tolerated	[89,90,91,92,93,94,100]
			Lanreotide	30 mg lanreotide sc once per month	129 mg/m depending on treatment response	Good results even comparable to surgery for early infants	[95,96]
			Pasireotide	0.15 mg 2 t/d		Better results in glycaemia control than lanreotide and octreotide; potentially a solution to avoid total pancreatectomy	[101,102]
Diabetic macular oedema	Different treatment schemes and strategy depending on patient condition and treatment availability	Many patients do not respond to treatments; some treatments may have unexpected complications or interfere with physiological cell function	SS	0.1% 2 t/d topical administration		Retinal arteriolar and venular dilation	[131]
			Octreotide	20 mg im	5000 mg sc per d for 15 m	From no effect to disease regression and reduction of haemorrhagic risk	[123,124,125,126,127,129,130,133]
			Lanreotide	90 mg sc 1/m for 1 y		Reduction of cystoid changes and foveal thickness; reduction of macular oedema	[132,134]
Graves orbitopathy	Glucocorticoids (local/systemic); monoclonal antibodies; immunosuppressants; surgical approaches	While this condition is usually treatable and self-limiting, chronic or fibrotic forms may be resistant to treatment; some immunosuppressants or monoclonal antibodies may have severe adverse effects	Octreotide	20 mg im 1/m for 4 m	100 μg sc 3 t/d for 3 m	No significant improvement to significant improvements in various parameters	[159,160,161,162,163,164,165]
			Lanreotide	0.04 g im 1/d for 2 w	30 mg im 1/2 w for 12 w	No significant improvement to statistically significant improvement	[141,166,167]
Angiodysplasias and GI bleeding	Surgical or pharmacological intervention depending on bleeding localization and volume; oestrogens and progesterone commonly used	Recurrent bleedings in spite of surgery; limited effect of pharmaceuticals	SS	100 μg sc 2 t/d for 26 m	>250 μg IV in 24 h	No effect to almost total control of bleeding	[187,192,193,194,195,196,197,198,199,206,207,208,210,215,223]
			Octreotide	50 μg/h IV cont. for 5 d	500 μg o IV for 2 suc. times and then 250–300 μg sc 2/d for 4 m	Bleeding control (equally or more effective than other schemes)	[201,202,203,204,205,209,211,214,216,217,219,220,221,222,224,226]
			Lanreotide	60 mg sc every 28 d for 6 m min.	9 mg sc every 28 d for 6 m min.	Improvement of anaemia and reduced healthcare costs	[227]
			Pasireotide	60 mg im every 28 d for 6 m		Transfusion requirements in patients with recurrent bleeding was reduced	[230]
			Vapreotide	50 μg IV bolus followed by continuous infusion of 50 μg/h for 5 d		Vapreotide may be useful but it did not significantly affect short-term outcomes	[228]
Acute pancreatitis	No specific treatment; pain medication and antibiotics prescribed as needed	Not all patients respond to treatment, especially if the underlying cause cannot be treated	SS	3.5 μg/kg/h IV cont. perfusion for 10 d	100–250 μg/h for 5 d	From slight improvement of biochemical markers to reduction of mortality and/or need for surgery	[242,243,244,245,246]
			Octreotide	0.5 μg/kg/h cont. IV infusion for 48 h	500 mg sc 3 t/d (24 h)	From negligible effects to progression prevention, reduction of sepsis and effective ERCP prophylaxis	[247,248,249,250,251,252,254,255,256,257,258,259]
			Lanreotide	30 mg im 1/d for 5 d		Decrease of pain after refeeding	[260]
Dumping syndrome	Proper diet; administration of viscosity modifying agents or acarbose for early and late dumping syndrome respectively	Potential failure of dietary or pharmacological treatment	SS	250–300 μg IV		Suppression of diarrhoea but pain on dumping provocation tests	[267]
			Octreotide	50 μg sc	500 μg sc 3 t/d for 3 d	Remission of symptoms to improvement of biochemical markers and quality-of-life	[268,269,270,271,272,273]
			Lanreotide	90 mg sc 1/m for 3 m		Successful management of symptoms but not quality-of-life improvement	[276]
			Pasireotide	300 μg 3 t/d for 2 w		Successful resolution of symptoms	[274,275]

SS = somatostatin, h = hours, d = days, w = weeks, m = months, sc = subcutaneously, im = intramuscular, IV = intravenous.

**Table 8:** Summary of SS and its analogues in their uses as second-line or alternative treatment/management agents, organized per pathology and mechanism.

Agent	Pathology	Beneficial Effects/Mechanisms of Action
SS	Diabetic macular degeneration	Retinal alveolar and retinal dilation
	Angiodysplasias & GI bleeding	Bleeding reduction/cessation; reduction in requirements for surgical intervention; reduction of rebleeding risk; reduction in post-endoscopic increase in hepatic venous pressure gradient
	Acute pancreatitis	Improvement in biochemical markers; reduction of complications; slower evolutions of pancreatic lesions; reduction in need for surgery; decreased hospitalisation length; reduction in mortality; decreased rate of pancreatic sepsis
	Dumping syndrome	Suppression of diarrhoea
Octreotide	Congenital hyperinsulinism	Reduction of blood insulin levels and normalisation of glucose levels; possible remission in some cases
	Diabetic macular degeneration	Reduction of IGF-1 levels; cessation or regression of retinal revascularisation; delay of disease progression; reduction of risk of dense vitreous haemorrhages; increased visual acuity; disappearance of cystoid changes
	Graves orbitopathy	Significant control/improvement of exophthalmos; improvement in proptosis, diplopia, and soft tissue involvement; improvement in eyelid fissure width; potential improvement in CAS
	Angiodysplasias & GI bleeding	Bleeding reduction/cessation; reduction of rebleeding risk; reduction of mortality; improvement of anaemia; reduction in transfusion needs
	Acute pancreatitis	Improvement in prognostic markers; reduction in mortality and complications; prevention of progression to severe acute pancreatitis; decrease in pancreatic oedema; effective prophylaxis for ERCP-induced pancreatitis
	Dumping syndrome	Suppression of diarrhoea; improvement of reactive hypoglycaemia; stabilisation of plasma insulin and GIP levels; increased in body weight; improvement in quality-of-life
Lanreotide	Congenital hyperinsulinism	Reduction of blood insulin levels and normalisation of glucose levels
	Diabetic macular degeneration	Reduction of cystoid changes and foveal thickness; reduction of macular oedema
	Graves orbitopathy	Significant improvement of exophthalmos;
	Angiodysplasias & GI bleeding	Improvement of anaemia; reduction in transfusion needs
	Acute pancreatitis	Reduction of pain after refeeding
	Dumping syndrome	Successful resolution of early dumping syndrome symptoms
Pasireotide	Congenital hyperinsulinism	Maintenance of euglycemia and potential avoidance of surgery
	Angiodysplasias & GI bleeding	Reduction in transfusion requirements
	Dumping syndrome	Improvement in dumping syndrome-associated biochemical markers; successful symptom resolution
Vapreotide	Angiodysplasias & GI bleeding	Potential effect in reduction of GI-associated bleeding

SS = somatostatin, GI = gastrointestinal, IGF = insulin-like growth factor, ERCP = endoscopic retrograde colangio-pancreatography, GIP = gastric inhibitory peptide, CAS = Clinical Activity Score.

### 8.1. SS Analogues in the Treatment of Congenital Hyperinsulinism: Potential and Complications

Conservative treatment has been proven feasible, with remission being possible at a mean timeframe of 49 months for SS analogue treatment. The risk of persistent growth retardation is low at about 5% [280]. Some severe adverse effects have been reported, such as hepatitis in an infant [281,282] necrotizing enterocolitis [99,283,284], rarely in a fulminant form [97]. In one infant, a paradoxical octreotide-induced hyperglycaemia and bradycardia during subtotal pancreatectomy was noted [285]. Of particular note is the successful treatment of a patient with a combination of octreotide and nifedipine [286]. We must note that octreotide’s effectiveness may be lost in the long-term due to tachyphylaxis [89,287], which can, however, be circumvented with proper temporal dose distribution [288]. From the studies recorded herein, all of them reported results in favour of SS analogues, which were better tolerated compared to other treatment schemes [94], whereas in certain cases, it was possible to avoid or decrease the percentage of resected pancreas [101,102]. We therefore conclude that SS analogues, with proper dosage and administration, can even be used as first-line treatment, with less severe adverse effects, compared to diazoxide, in patients wishing to avoid surgery or when surgery cannot be performed.

### 8.2. SS and Its Analogues in the Management of Ocular Pathologies Associated with Chronic Diseases

#### 8.2.1. Diabetic Retinopathy and Macular Degeneration

SS and its analogues appear to be promising candidates for developing new treatment strategies, even if there are fewer results available compared to other uses of SS analogues. Several mechanisms have been proposed by which SS and its analogues are believed to work against diabetic macular degeneration, all centered around the inhibition of GH and IGF-1 production, which in turn leads to VEGF, basic fibroblastic growth factor, endothelial growth factor (EGF), and platelet-derived growth factor inhibition [134].

The available research results appear to be positive, with two notable exceptions being early trials [125,126]. Not all of the performed studies have included large patient groups or were randomised, with no one being double-blinded, which would increase their statistical significance and validity. While the nature of ocular pathologies frequently predisposes to topical treatments, most SS analogues have been systemically administered. Of note, the only application of SS was performed locally with favourable results [131].

#### 8.2.2. Graves Orbitopathy (TED)

The therapeutic effects of SS analogues are most probably based on the IGF-1 immunoreactivity exhibited by the implicated tissues [157], and on inhibition of lymphocyte proliferation and activation [164]; thus, pro-inflammatory cytokine production (like TNF-α, IFN-γ, IL-1, TGF-3) is inhibited [289].

The results from available clinical trials are mixed at best. Some early research documented a significant therapeutic benefit [159,160,162,163,166], but with the exception of the penultimate one, they were non-randomised studies, and all had a very small sample size. On the other hand, amongst the larger, randomised, double-blind trials [141,164,165,167], only one documented a significant improvement in proptosis, even if the disease activity was not reduced [164]. While the reported results are not generally encouraging, there is a role, both diagnostic and predictive and therapeutic, for SS analogues, especially if newer drugs, with higher affinity for SSTRs, become available [119,146].

### 8.3. SS and Its Analogues in the Treatment of GI Bleeding: Combination with Existing Therapeutic Modalities, and Treatment Results

SS and its analogues have been tried in the cases of GI bleeding resulting mostly from peptic ulcers, varices, and angiodysplasias. In some cases, there have been some therapeutic failures [199,221], or the effect was less than that of other more interventional measures [203]. In other cases, octreotide was found to be as effective as terlipressin [207,223] and sclerotherapy [209,210], and, sometimes, while the bleeding itself was not reduced, hospital stay and the need for additional therapeutic measures were diminished, thus reducing the strain on the healthcare system [216,226,230], in the latter case using pasireotide. With the exception of these studies, others herein recorded have reported results in favour of SS and its analogues. One attempt with vapreotide noted that it is potentially useful and should be investigated further [228]. Octreotide injections were proven to be able to cause transient reductions in portal vein blood pressure and azygos vein blood flow, but continuous infusions seem to cause desensitization and tachyphylaxis [290].

Regardless of the success of pharmacological treatment, in most cases, some type of intervention may be required to address the cause of the bleeding. In the case of angiodysplasias, surgical treatment comprises different modalities, such as argon plasma coagulation, electrocoagulation, and laser photocoagulation [291] or local haemostatic injections [292]. Endoscopic methods, while successful, have their limitations, and in about 30%, there is some degree of rebleeding [293]. Oesophageal varices, frequently encountered in the context of cirrhosis, are also treated endoscopically, requiring relatively frequent follow-ups thereafter [294]. However, the use of SS and its analogues can mitigate bleeding, allow for easier visualization of the bleeding site, and reduce post-surgical complications and treatment requirements.

It is evident that many cases of GI bleeding are associated with cirrhosis and, in turn, portal hypertension [295,296]. As it had been observed early on that SS reduces splanchnic blood flow with minimal effects in systemic circulation, it was thought that it could be effective in patients with portal hypertension. Probably the first study of its effect on cirrhotic and non-cirrhotic patients was performed in 1986 [297] and noted a decrease in portal bleeding when they administered 250 μg SS IV over 30 s. A significant decrease in portal vein pressure was noted in the subsequent trial of Yang et al. [298], who demonstrated this decrease both after SS and octreotide administration; this trial was significant in that it was both randomised and double-blinded, therefore having greater validity. In the same year, however, another randomised double-blinded trial did not find any difference in portal hypertension between the control and test group, when SS was administered along with terlipressin [299]. Two more recent trials, again using SS, in 2013 [300] and 2019 [301] documented positive results, leaving few studies with negative results [299].

A number of notable clinical trials, all randomised except one [302], were performed using octreotide for the same problem [298,302,303,304,305,306]. Some [302,305,306] did not detect any noticeable reduction in hepatic venous pressure, while others [298,303,304] have indicated that there is therapeutic potential. In those cases where no appreciable effect was noted, the dose and method of administration appear to play a role [303,307,308,309,310]. Finally, a few research efforts have focused on lanreotide; randomised double-blinded trials [311,312] and non-randomised studies [313] noted a significant decrease in portal vein pressure. Combined with the results on GI bleeding cases, we conclude that SS and its analogues may be used both to reduce GI bleeding and portal hypertension when these two clinical entities are associated, both on their own and as adjuvant treatments to endoscopic processes.

### 8.4. SS and Its Analogues in the Treatment of Acute Pancreatitis: Potential for a First-Line Treatment?

Regarding SS analogues in the management of acute pancreatitis, the results are generally in favour of their use. While a couple of trials did not record any significant beneficial results when using octreotide [243,249,250,251,258], most other researchers mention some type of positive result, for example, improvements in biochemical markers and/or reduction of complications [242,245,246,247,255,256,257]. Others reported a delay in the evolution of the pancreatic lesions [244,259], mentioned the potential of octreotide as prophylaxis against ERCP-induced acute pancreatitis [254], and demonstrated that lanreotide can reduce pain during refeeding after therapy [260].

Of particular interest is a case where SS was used to treat pancreatitis induced by systemic lupus erythematosus [314], showcasing its potency even in patients with complex pathological profiles. Another rare case study described a patient in whom acute pancreatitis developed as a complication of aortic dissection. The patient was treated with an injection of somatostatin, ultrasound-guided puncture drainage, and jejunal nutrition tube placement and discharged after a 30-day period [315]. A randomised controlled trial, comparing high-dose versus low-dose octreotide treatment in acute pancreatitis patients, found that high doses of octreotide with 48 h of acute pancreatitis onset could reduce the risk of developing its severe form, or at least partially attenuate it, by reducing IL-6 and TNF-α levels [316].

While there is a relative lack of big data analysis on the use of enzyme inhibitors in acute pancreatitis patients, the retrospective cross-sectional analysis of Sun et al. [317] indicated that perhaps SS is more effective than octreotide, even though, as noted by the authors themselves, a number of limitations existed in their study. In cases where patients would develop acute pancreatitis following SS analogue administration—for another therapeutic indication—it has been proposed that lanreotide autogel could be used with similar effects but lower risk of acute pancreatitis, should the same patients need another administration of SS analogue in the future [318]. Despite it being evident that SS analogues are not useful in all clinical situations involving acute pancreatitis, they can be used, being generally well-tolerated, in the absence of other effective pharmacological management solutions.

### 8.5. SS Analogues as Potential Alternatives in the Treatment of Dumping Syndrome

All the trials presented in detail in the relevant subsection were more or less successful. In addition, there have been other trials and cases, where octreotide was used to manage the symptoms of dumping syndrome with varying degrees of success [319,320,321,322], but not all the relevant data are available.

A particular case was recorded by Sato et al. [323], where octreotide was administered in a 47-year-old woman who, 10 years before, had undergone distal gastrectomy with Billroth I reconstruction for early-stage gastric cancer; octreotide prevented most of the symptoms of early dumping syndrome, including postprandial tachycardia, but caused postprandial hyperglycaemia. Following an OGTT test, GLP-1 and glucose-dependent insulinotropic polypeptide (GIP) were completely suppressed, but no change in VIP was recorded—the hormone generally responsible for early dumping syndrome; it was hypothesized that, at least in that particular patient, there was a possible contribution of incretins to her early dumping syndrome. Another interesting case report, regarding a woman who underwent a minimally invasive esophagectomy for a moderately differentiated squamous cell carcinoma of the oesophagus, and presented with symptoms suggestive of dumping syndrome, after about two years, was published in 2017 [324]. A peculiarity of the case was that she was initially treated with octreotide, then with lanreotide, which actually aggravated her condition, and finally with pasireotide, which had the best results when combined with diazoxide. This last treatment scheme has proven successful in the long-term and may provide a potential solution in late dumping syndrome, which is generally more difficult to treat compared to early forms [324].

### 8.6. Other Applications of SS Analogues and Future Potential

A minor application of SS analogues is in the treatment of digestive and lymphatic fistulas, which are relatively frequent complications of surgeries, and are associated with significant morbidity and mortality [325,326,327]. Being able to inhibit gastric secretion and motility, and to reduce thoracic duct lymph flow rate, both octreotide and lanreotide have been shown to promote the closing of fistulas, even if they do not always appreciably decreased mortality [119]. They are an effective adjuvant treatment, with the best results being achievable within 10 days of surgery [328,329,330,331].

Another rare use of SS analogues is in patients suffering from polycystic kidney disease (PKD), which may be complicated with polycystic liver disease (PLD). PKD is a congenital disease, transmitted by an autosomal dominant pattern [332]. Cyst formation is mediated by the adenylyl cyclase pathway, a cAMP-dependent signalling pathway [333]. Via their action on SSTR_2_, SS analogues can suppress this pathway in renal and hepatic cells, thereby hindering cyst formation [119]. About 20 years ago, a notable research effort in that direction is the clinical trial of Ruggenenti et al. [334]; since then there have been quite a number of studies on the matter, and based on recent meta-analyses, SS analoguescan be used to at least reduce the rate of renal and hepatic volume increase, although more research is required to determine the cost–benefit ratio of that particular treatment avenue [335,336].

SS analogues have also been tried in the treatment of refractory chronic diarrhoea, a term used to describe a diarrheal episode of more than 14 days that does not respond to specific antimicrobial therapy or nonspecific antidiarrheal therapeutic measures; they are different causes, each one presenting with therapeutic particularities [337]. Based on existing data, it seems that there are cases where SS analogues meaningfully improve the symptoms and patients’ quality of life [338,339], but further research is required [340,341,342]. Finally, there is evidence to support the application of octreotide in the management of hepatorenal syndrome [343], in association with albumin and other drugs [344], and in the management of orthostatic hypotension, where it raises systemic blood pressure by causing splanchnic vasoconstriction [345,346].

The overwhelming majority of evidence for the effects of SS and SS analogues in different pathologies concerns adult patients, with the exception of cases of congenital hyperinsulinism. For most of the pathologies herein presented, uses of SS analogues, predominantly octreotide, are recorded in paediatric patients, even though the dosages are different, with no precise guidelines in place, and clinicians should be wary of side and adverse effects [347] (and references therein). Perhaps the only application specific to paediatric patients is cases of chylothorax and/or chyloperitoneum [98,348,349,350,351,352,353,354,355,356,357], and primary intestinal lymphangiectasia (Waldmann’s disease) [358], a rare disorder characterized by dilated intestinal lacteals resulting in lymph leakage into the small bowel lumen, leading to protein-wasting enteropathy [359].

Apart from SS analogues approved for clinical use, there have been some other analogues under development, in various stages of testing. Vapreotide has been tested both in vitro and in vivo [53,54,228,229,360,361,362,363,364,365,366,367,368], and, based on current evidence, it seems to have a promising potential in the field of oncology [369,370,371], apart from the few applications reported in the GIT bleedings subsection. Another analogue, veldoreotide, has not yet been tried clinically [372]; finally, seglitide has seen few applications [46,361], after some limited in vitro testing, even though it has been synthesized since the 1980s.

#### 8.6.1. Use of SS Analogues in the Context of Infectious Diseases

Regarding vapreotide specifically, an interesting application was examined in 1992, in a clinical trial in France, involving 34 AIDS patients with chronic diarrhoea, who were unresponsive to any available medication at the time. Cryptosporidiosis, i.e., infection with *Cryptosporidium parvum*, was diagnosed in 21 out of the final 30 patients evaluated. It was found that vapreotide was effective only in those patients whose diarrhoea was not caused by cryptosporidiosis [360]. Cryptosporidiosis is transmitted via the oral–faecal route, and its pathogenic potential was recognized after the 1980s, especially in immunosuppressed patients, either medically or due to human immunodeficiency virus (HIV) [373]. Notably, there is no effective treatment for cryptosporidiosis, with only prevention measures being available [374].

Apart from *C. parvum* infections, SS may be of interest in cases of cysticercosis, a parasitic infection caused by *Taenia solium* cysts. The parasite itself usually causes little to no inflammation, but upon dying it triggers a severe granulomatous inflammation; in late murine granulomas, somatostatin is expressed in the involved cells, at a higher rate compared to earlier stages [375]; based on experiments in mice, it is believed that SS downregulates granuloma formation, and therefore SS analogues may represent a viable treatment alternative in such cases [376]. While it is difficult to quantify the burden of disease for cysticercosis, due to a number of diagnostic complexities and the limitations of existing observational studies [377], it represents a potentially lethal disease in endemic areas [378].

Notably, octreotide has been tried as a treatment in mice infected with *Schistosoma mansoni*. Again, SS was found to be expressed in associated granulomas, in murine models, and it is hypothesized that SS analogues may protect against schistosomiasis-induced inflammation by regulating lymphokine levels [379,380]. Apart from that, SSTR_2_ seems to be expressed in T lymphocytes in hepatic granulomas, in murine models infected with *S. mansoni* [381]. Schistosomiasis is characterized by a notable burden of disease in certain areas [382,383]. It is currently manageable practically only by praziquantel administration, a useful drug but with a number of limitations [384]. Since vector control strategies are not always effective or practical, this parasitic disease continues to represent an endemic health hazard, necessitating the development of novel treatment methods [385]. The potential applications of SS analogues in the case of parasitic infections is important, given that granulomas are frequent in parasitic infections [386], and that antiparasitic medications often have potentially severe adverse effects [387,388]. It should also be mentioned that octreotide was tested in the past as a treatment for quinine-induced hyperinsulinemia in both healthy volunteers and patients with malaria [389].

Another interesting aspect concerns *Helicobacter pylori*, a pathogen associated both with increased gastric ulcer [390,391] and gastric cancer incidence [392]; despite available antibiotic treatments, some *H. pylori* strains exhibit worrisome resistance profiles [393]. It is known that *H. pylori* lowers the density of δ pancreatic cells, therefore increasing gastrin production as SS levels decline; this may be an explanation for *H. pylori* infection being a risk factor for gastric ulcer development [394]. Given the gut microbiota disruption caused by *H. pylori* [395], which also further influences the nervous system [396], more research on the precise role of SS in *H. pylori* infections should be performed.

While there is little evidence on the interplay between somatostatin and infections, excluding the particular case of *H. pylori*, there are some tentative indications that indicate that somatostatin levels are affected in some cases. There seems to be SS and SSTR expression in certain cells of the immune system, although their precise role has not been elucidated [397]; however, it is known that neuropeptides, such as SS, are involved in immune system regulation [398], and, in turn, inflammatory cytokines regulate SS levels in immune system cells [399]. The existence of SS on lymphocytes has been documented by a number of researchers [400,401]. The research on the effects of SS in the response of dendritic cells has led Kao et al. [402] to suggest that SS administration may be useful in treating immune-mediated diseases of the stomach.

#### 8.6.2. SS and Its Analogues in Modulation of Inflammatory and Immune Responses

More recently, it was shown, albeit in a mouse model, that SS can inhibit the NF-κB pathway, and thus protect against intestinal barrier dysfunction during sepsis [403]; investigations specifically into the role of SS in intestinal inflammation had also been previously carried out [404,405,406]. Apparently, in the short term, SS and octreotide may be able to modulate the progression of liver fibrosis [407]. There has been evidence that SS inhibits inflammatory response [408] and oxidative damage [409], therefore having a potential cytoprotective effect [410]; this may prove useful in cases such as heavy metal intoxication, where oxidative damage is one of the principal avenues of damage [411]. The potential of SS to downregulate inflammatory mediators has been proven by different research efforts in the past [412,413]. Since it is evident that, at least in the handful of examined cases, SS, and therefore its analogues, can reduce pathogen-induced inflammation, a future avenue of research could concern the incorporation of suitable SS analogues into 3D printed biomaterials, used either for tissue replacement [414,415] or in osseous reconstruction [416].

Based on the immunomodulatory effects of somatostatin, Zhang et al. used octreotide to treat the symptoms of systemic lupus erythematosus (SLE) in a clinical trial comprising 26 patients, some on octreotide and some on corticotherapy, and 11 healthy individuals [417]. It was found that octreotide decreased peripheral mononuclear cells and proinflammatory cytokine production, and was correlated with decreased SLE disease activity index (SLEDAI) values [417]. In the early years of the 21st century, some research was performed towards the direction of using somatostatin analogues in immune system regulation [418] and associated disorders [419], but there has not been any significant follow-up in this direction. Given the difficulties and challenges in the treatment of autoimmune pathologies [420,421,422,423], and their oftentimes multifactorial nature [424,425,426], SS analogues could potentially be considered for therapeutic applications following the necessary trials. Moreover, there exists a number of phytochemicals with antioxidant, anti-infectious, and anti-inflammatory potential, such as kaempferol [427,428], capsaicin [429,430], pinosylvin and other stilbenoids [431,432], curcumin [433,434], thymol [435], and piperine [436,437], which could be administered along with SS analogues to hopefully improve disease markers and quality of life in those patients.

When considering all the discussed aspects of SS levels and their potential significance either as disease markers, or, more importantly, as indications for treatment, the possibility of administering SS analogues directly at the affected sites, in cases of infections, should be looked into. Indeed, in recent years, the delivery of drugs via lipid particles or nanoparticles has been used for the delivery of a number of drugs [438,439,440,441,442,443,444].

#### 8.6.3. Induction of Endogenous SS Production

Irrespective of the particular pathology that SS analogues are used to treat, either neoplastic or not, apart from SS analogue administration, the induction of endogenous somatostatin production might be considered. This can be achieved by acupuncture, and there exists literature data to support the alteration of serum hormone and protein levels in both humans and animals, following acupuncture [445,446,447,448,449,450,451]. Specifically for SS, electroacupuncture has been found to increase serum SS levels and possibly influence SSTR expression in rabbits [452]. Electroacupuncture and moxibustion could also potentially affect serum SS levels in humans [453,454]; in turn, it is believed that serum SS levels could influence the therapeutic results of electroacupuncture [455,456]. As a potential research avenue, it could also be explored whether SS or SS analogue administration can modify the electrical parameters in acupuncture points [457,458], and potentially modify the treatment outcome.

#### 8.6.4. Somatostatin Receptor Antagonists and Their Potential Uses

Another aspect of particular interest is the potential for diagnostic and clinical use of SSTR antagonists. Tested applications comprise the identification of thyroid cancer [459] and other neoplasias [460]; it is also possible to use them as a tool to evaluate response to treatment [461]. SSTR agonists are much more internalized compared to SSTR antagonists, and are thus less valuable from an imaging perspective [462]; in neuroendocrine pathologies in particular, it is believed that SSTR antagonists are able to bind to a higher number of SSTR receptor conformations, compared to agonists [463,464]. On the treatment front, only one major avenue has been explored in humans [465], although a number of different compounds are being tested in mice, and the relevant results are promising [466,467,468,469,470,471,472,473,474,475,476,477].

## 9. Conclusions

Somatostatin and its analogues represent viable therapeutic solutions for a host of different non-neoplastic pathologies, but their efficacy and supporting evidence vary considerably between various conditions. In most situations, they should be regarded primarily as second-line choices, complementary, or rescue therapies, especially in patients where standard treatment is not tolerated, is ineffective, or contraindicated. The literature supports the potential use of somatostatin and its analogues for cases of congenital hyperinsulinism, gastrointestinal bleeding, acute pancreatitis, portal hypertension, dumping syndrome, and some ocular disorders. However, the evidence is heterogeneous and limited as their clinical role appears to be influenced by disease mechanisms as well as receptor and agonist profiles. Their potential to modulate inflammation, oxidation, and the immune system may also give rise to interesting new applications in the field of infectious diseases and in pathologies where oxidative damage is the main pathophysiological mechanism. Further studies are needed to better define the category of patients most likely to benefit from somatostatin analogues within specific treatment algorithms.

## Data Availability

No new data were created or analyzed in this study. Data sharing is not applicable to this article.

## Abbreviations

The following abbreviations are used in this manuscript:

ACTH	Adrenocorticotropic hormone
AIDS	Acquired immunodeficiency syndrome
APTS	Acute pancreatitis with somatostatin
ATP	Adenosine triphosphate
B	Blinded
cAMP	Cyclic adenosine monophosphate
CAS	Clinical Activity Score
CCK	Cholecystokinin
CNS	Central nervous system
CO	Crossover
DB	Double-blinded
EGF	Endothelial growth factor
ERCP	Endoscopic retrograde cholangiopancreatography
EST	Endoscopic sphincterotomy
GAVE	Gastric antral vascular ectasia
GH	Growth hormone
GHRH	Growth-hormone-releasing hormone
GI	Gastrointestinal
GIP	Glucose-dependent insulinotropic polypeptide
GIT	Gastrointestinal tract
GLP-1	Glucagon-like peptide-1
GRPC	G protein-coupled receptor
HCC	Hepatocellular carcinoma
HIV	Human immunodeficiency virus
IFN-γ	Interferon gamma
IGF-1	Insulin-like growth factor I
IL-1	Interleukin 1
IL-6	Interleukin 6
im	Intramuscular
iv	Intravenous
Kir3.x	Inward rectifier current K+ channels
LA	Long-acting
LAR	Long-acting release
LDH	Lactate dehydrogenase
NET	Neuroendocrine tumour
NF-κB	Nuclear factor kappa-light-chain-enhancer of activated B cells
NME	Necrolytic migratory erythema skin rash
NR	Non-randomised
OGTT	Oral glucose tolerance test
OLE	Open-label extension
PGDF	Platelet-derived growth factor
PKD	Polycystic kidney disease
PLD	Polycystic liver disease
PR	Prolonged-released
PTX	Pertussis toxin-sensitive
R	Randomised
sc	Subcutaneous
SLE	Systemic lupus erythematosus
SLEDAI	SLE disease activity index
SR	Slow-release
SRIF14	Somatotropin Release-Inhibiting Factor-14
SRIF28	Somatotropin Release-Inhibiting Factor-28
SS	Somatostatin
SSTR	Somatostatin receptor
TED	Thyroid eye disease
TGF-3	Transforming Growth Factor-BETA 3
TNF-α	Tumour necrosis factor alpha
TSH	Thyroid stimulating hormone
VEGF	Vascular endothelial growth factor
VIP	Vasoactive Intestinal Peptide
VWD	von Willebrand’s disease
WBC	White blood cell
